# Functional interaction between macrophages and hepatocytes dictate the outcome of liver fibrosis

**DOI:** 10.26508/lsa.202000803

**Published:** 2021-01-29

**Authors:** Min Xie, Ren Hui Chia, Dan Li, Fanny Xueting Teo, Christian Krueger, Kanaga Sabapathy

**Affiliations:** 1 Division of Cellular and Molecular Research, Humphrey Oei Institute of Cancer Research, National Cancer Centre Singapore, Singapore, Singapore; 2 Cancer and Stem Cell Biology Program, Duke-NUS Medical School, Singapore, Singapore; 3 Department of Biochemistry, Yong Loo Lin School of Medicine, National University of Singapore, Singapore, Singapore; 4 Institute of Molecular and Cellular Biology, Singapore, Singapore

## Abstract

Hepatocytes and liver-resident macrophages known as Kupffer cells (KCs) are key cell types involved in liver fibrosis. The transcription factor c-Jun plays a fundamental role in regulating hepatocyte and macrophage functions. We have examined c-Jun’s role in the functional interaction of these cells during liver fibrosis induced by carbon tetrachloride. While hepatocyte-specific *c-jun* deletion led to increased fibrosis, the opposite outcome was observed when *c-jun* was deleted in both hepatocytes and KCs. Molecular analyses revealed compromised cytokine gene expression as the apical event related to the phenotype. Yet, purified hepatocytes from both mouse cohorts showed similar defects in cytokine gene expression. However, we noted increased macrophage infiltration in the absence of c-Jun in hepatocytes, which when chemically depleted, reversed the phenotype. Consistently, *c-jun* deletion in KCs alone also led to reduced fibrosis and cytokine gene expression. By contrast, *c-jun* deletion in hepatocytes and KCs did not affect the resolution phase after fibrotic injury. These data together demonstrate a pro-fibrogenic role for c-Jun in hepatocytes and KCs that functionally interact to regulate liver fibrosis.

## Abstract (precis)

Xie et al demonstrate opposite outcomes on liver fibrosis due to deletion of c-Jun in hepatocytes alone or along with Kupffer cells, highlighting the functional interaction between these cell types in regulating the fibrotic process, dictated by c-Jun.

## Introduction

Fibrosis of the liver is a process of wound healing that is activated upon injury to the liver. It occurs under a variety of contexts including viral infection, alcohol overconsumption and fatty liver disease arising from high-fat diet (Trautwein et al, 2015; Koyama & Brenner, 2017). Chronic stimulation exacerbates the wounding process, leading to severe scarring. This is often seen in the lead-up to the formation of hepatocellular carcinoma (HCC), where stimulation with alcohol or high-fat diet could lead from fibrosis, to cirrhosis and consequently, HCC formation (Michelotti et al, 2013; Koyama & Brenner, 2017). However, when the stimuli are removed, the scars are resolved by proteases (Ramachandran et al, 2015). Several cell types are involved in orchestrating liver fibrosis. These include the hepatocytes and liver-resident macrophages known also as Kupffer cells (KCs) that produce fibrogenic cytokines to initiate the fibrotic process of activating the hepatic stellate cells (HSCs). Activated HSCs then produce collagens which are deposited as scars. All three cell types contribute to the secretion of chemokines such as CCL2 that leads to the recruitment of bone-marrow-derived monocytes, which develop into Ly-6C^+^ macrophages that further promote liver injury, leading to further HSC activation (Ju & Tacke, 2016). Depletion of KCs by several means has been shown to attenuate liver fibrosis (Guillot & Tacke, 2019), suggesting a critical role for this cell type in liver pathogenesis.

Many signaling pathways have been implicated in regulating liver fibrosis, including hedgehog, JNK, and IKKβ, amongst others (Sun & Karin, 2008; Seki et al, 2012; Machado & Diehl, 2018). Of these, the JNKs have been shown to regulate liver fibrosis varyingly. Global JNK1 deficiency or absence of JNK1 in hepatocytes alone have been shown to differentially affect fibrosis induced by carbon tetrachloride (CCl_4_), by choline-deficient diet treatment and by bile-duct ligation (Kodama et al, 2009; Zhao et al, 2014; Cubero et al, 2016). In the case of global JNK1 deficiency, bone marrow–derived cells were thought to be contributory in one case as hepatocyte-specific deletion of JNK1 did not significantly affect fibrosis in that study (Kodama et al, 2009), whereas HSCs rather than bone-marrow derived cells were thought to be causal in another study (Zhao et al, 2014). Moreover, hepatocyte-specific JNK1 deficiency was found not to significantly affect fibrosis (Zhao et al, 2014; Cubero et al, 2016), despite higher levels of liver injury as determined by elevated aspartate aminotransferase (AST) levels. These studies together highlight that the JNK signaling pathway regulates liver fibrosis through multiple cell types in the liver likely through complex interactions.

c-Jun is an integral member of the activator-protein (AP)-1 family of transcription factors and a direct substrate of the JNKs, mediating many of the stress-regulated responses (Eferl & Wagner, 2003; Shaulian, 2010). It regulates a variety of cellular processes such as apoptosis, proliferation and differentiation, thereby regulating organismal physiology (Eferl & Wagner, 2003; Shaulian, 2010). c-Jun is a critical regulator of liver functions in various contexts: it is required for embryonic liver development (Hilberg et al, 1993), is a limiting factor for hepatocellular carcinoma (HCC) formation induced by chemicals (Eferl et al, 2003), and is necessary for liver regeneration upon partial hepatectomy (Behrens et al, 2002). c-Jun expression is up-regulated in many liver conditions such as fibrosis and non-alcoholic steatohepatitis (NASH), both in hepatocytes and non-parenchymal cells (Schulien et al, 2019). A recent study showed that hepatocyte-specific deletion of c-Jun led to increased fibrosis induced by methionine/choline-deficient diet, whereas combined deletion in non-parenchymal cells ameliorated the response (Schulien et al, 2019). Independently, c-Jun has been deleted in macrophages which affected the pro-inflammatory response, reducing bone-destruction in an arthritis model (Hannemann et al, 2017). We have, therefore, investigated the role of c-Jun in hepatocytes and KCs in regulating liver fibrosis. Our results suggest that c-Jun promotes fibrosis through both these cell types, but in a coordinated manner.

## Results

### *c-jun* deletion in hepatocytes alone or together with KCs result in differential effects on liver fibrosis

**Figure S1.**
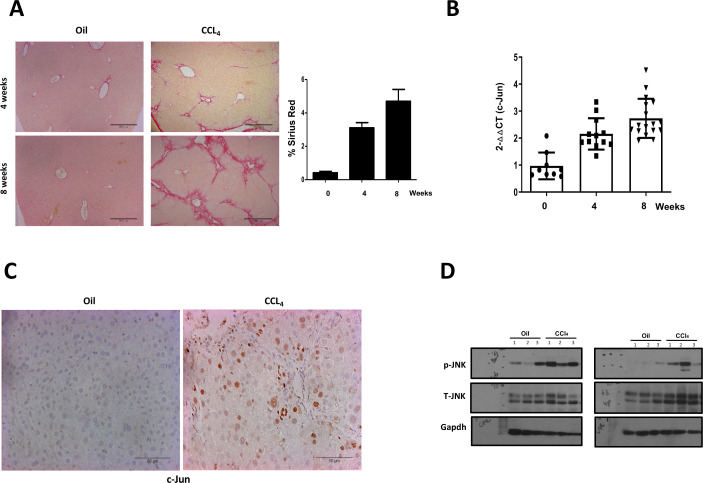
c-jun is activated by carbon tetrachloride-induced liver fibrosis. **(A)** Extent of liver fibrosis in wild-type mice after 4 or 8 wk of oil or CCl_4_ injection was determined by Sirius red staining (left panels). Liver sections from representative mice are shown (10× magnification), and quantified (right panel), as described in legends to Fig 1. n = 0 wks – 8; 4 wks – 10; 8 wks – 8. **(B)** Quantitative real-time-PCR of *c-jun* expression in livers was determined after CCl_4_ treatment for the indicated time periods. Each dot represents a liver from an individual mouse. Data represent mean ± SD. n = 0 wks – 9; 4 wks – 12; 8 wks – 17. **(C)** Expression of c-Jun was determined by immunohistochemical staining on whole liver sections, and representative images at 8 wk after CCl_4_ or oil injection are shown. **(D)** Expression of phopho (p)-JNK and total (T)-JNK was determined by immunoblotting using livers from three independent mice (1–3). Two independent experiments are shown with different sets of mice. The Gapdh blot was performed once for all lysates, and the blot shown here in Fig 1D left panel is the same as shown in Figs 1C and 2F, as the same set of lysates was used in these figures. Source data are available for this figure.

Experimental induction of liver fibrosis in mice by repeated CCl_4_ treatment leads to liver injury (Moles et al, 2014), assessable by Sirius red staining that marks the deposition of collagen in the fibrotic area, reflecting the extent of injury (Fig S1A). Continual CCl_4_ treatment was accompanied by a significant increase in the expression of *c-jun* in the liver (Fig S1B). Consistently and similar to published observations in humans (Schulien et al, 2019), c-Jun levels were also up-regulated in the liver (Fig S1C), concomitant to an increase in the expression of phosphorylated-JNK (Fig S1D), indicative of a plausible role for the JNK-c-Jun pathway in regulating the hepatic fibrosis process.

**Figure S2.**
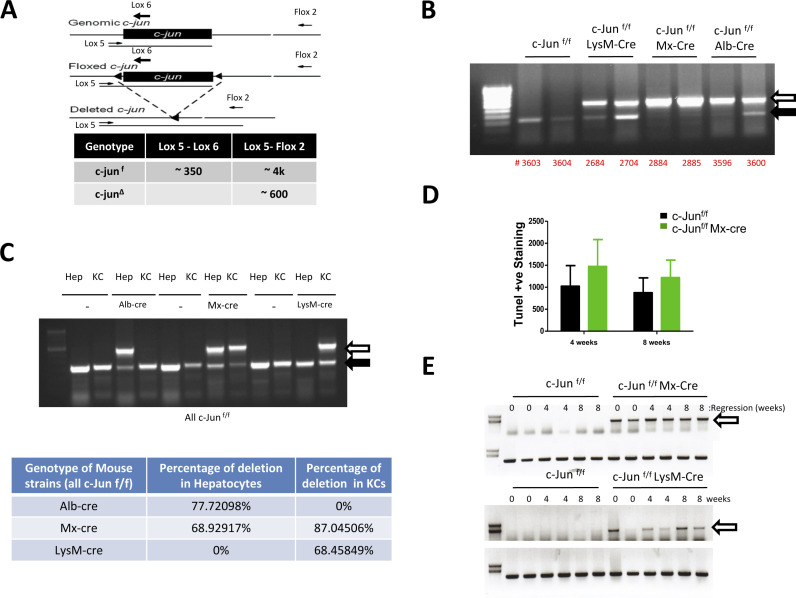
*c-jun* deletion in various mouse strains and isolated cells. **(A)** Schematic shows *c-jun* locus and the position of PCR primers used for genotyping, and the expected size of the PCR products (table). c-jun^f^ and c-jun^Δ^ represent *c-jun* locus in mice without or with cre in the various cell types. **(B, C)** Genotyping results using genomic DNA from whole livers (B) or purified hepatocytes and Kupffer cells (C) are shown. Solid arrow indicates wild-type *c-jun* locus, corresponding to 350 bp; and open arrows indicate deleted *c-jun* locus corresponding to 600 bp. **(B)** Representative results from two separate livers from independent mice are shown (B). **(C)** Extent of cre-mediated recombination of *c-jun* in purified cells from c-jun^f/f^ mice in the absence or presence of the various cre-expressing mouse strains is shown (C). **(C)** Percentage of *c-jun* deletion was determined by comparing the intensity ratio of the deleted band and the sum of both the deleted and undeleted bands and is shown in table (C). **(D)** Extent of cell death in livers from c-jun^f/f^ and c-jun^f/f^;Mx-cre mice was determined by TUNEL staining, using Millipore S7100. n = c-jun^f/f^ CCl_4_: 4 wk – 8; 8 wk – 8 c-jun^f/f^;Mx-cre CCl_4_: 4 wk – 8; 8 wk – 8. **(E)** Livers from two mice each for a time point from the regression experiments were used to show *c-jun* deletion in the indicated groups. Upper blot shows deleted *c-jun* locus as described above (open arrows) and lower blot shows the presence of the c-jun^f^ region in the locus as an internal control for loading.

**Figure 1.**
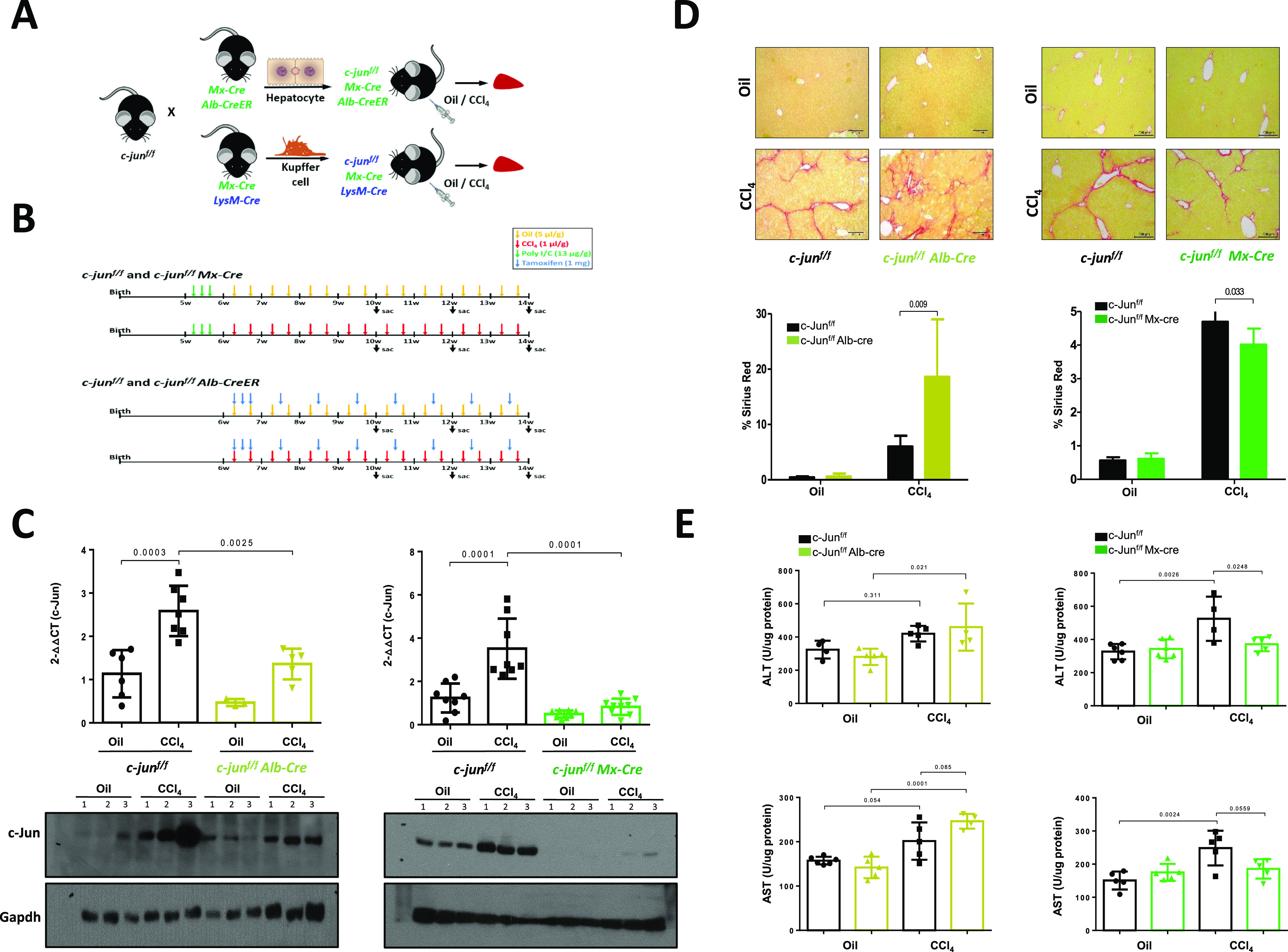
*c-jun* deletion in hepatocytes alone or together with Kupffer cells results in opposite effects on fibrosis. **(A, B)** Breeding scheme of mouse strains used in the study. **(A)** c-jun^f/f^ mice were crossed to the indicated strains of Cre-expressing mice to delete *c-jun* in the various cell types (A). **(B)** Mice were either treated with oil or carbon tetrachloride (CCl_4_) twice per week for 8 wk to induce fibrosis, and livers were harvested at 72 h after the last injection (B). Poly I:C or tamoxifen was injected as indicated to induce the expression of Cre in Mx-cre or to activate the Cre-ER in Alb-cre mice, respectively. **(C)** Expression of *c-jun* was determined by quantitative real-time PCR (upper panel) and by immunoblotting (lower panel) using whole livers from the indicated mice. Experiments were performed in duplicates and data represents mean ± SD. Each dot represents a liver from an individual mouse. n = c-jun^f/f^: oil – 6; CCl_4_ – 7; c-jun^f/f^;Alb-cre: oil - 3; CCl_4_ – 5; c-jun^f/f^: oil – 8; CCl_4_ – 8; c-jun^f/f^;Mx-cre: oil – 8; CCl_4_ – 9. Each number represents a mouse in the immunoblot analysis. Statistics was performed by two-way ANOVA. Multiple blots were run with the same lysates for detection with the various antibodies. The Gapdh blot was performed once for all lysates, and the blot shown here is the same as shown in Figs 2F and S1D left panel, as the same set of lysates was used in these figures. **(D, E)** Extent of liver fibrosis and damage after 8 wk of oil or CCl_4_ injection was determined by Sirius red staining (top panels), and representative pictures are shown (10× magnification) (D). Quantification of fibrosis is based on analyzing 20 randomly chosen fields from four individual liver lobes per mouse (lower panels). Data represent mean + SD. n = c-jun^f/f^: oil – 6; CCl_4_ – 7; c-jun^f/f^;Alb-cre: oil - 3; CCl_4_ – 6; c-jun^f/f^: oil – 8; CCl_4_ – 8; c-jun^f/f^;Mx-cre: oil – 8; CCl_4_ – 9. Statistics was performed by unpaired *t* test. **(E)** Extent of liver damage was evaluated by determine the levels of ALT and AST in the livers (E). Data represent mean + SD. n = c-jun^f/f^: oil – 4/6; CCl_4_ – 5; c-jun^f/f^;Alb-cre: oil – 5; CCl_4_ – 4; c-jun^f/f^: oil – 5/6; CCl_4_ – 5/6; c-jun^f/f^;Mx-cre: oil – 4/5; CCl_4_ – 5. Statistics was performed by two-way ANOVA. Source data are available for this figure.

Given the extensive analysis of the role of JNKs in liver fibrosis (Seki et al, 2012), we undertook a genetic approach by deleting c-Jun in hepatocytes and KCs to understand its role in the liver fibrotic process. We used the c-Jun floxxed (c-jun^f/f^) mouse strain in which the *c-jun* gene is flanked by loxP sites (Behrens et al, 2002) (Fig S2A), and crossed them to a variety of mouse strains expressing the Cre-recombinase under the following promoters: Mx1-cre (for deletion mainly in hepatocytes and hematopoietic cells including monocytes) (referred hereafter as Mx-cre), albumin-cre (for deletion in hepatocytes only), and lysozyme-M (LysM)-cre (monocyte-specific deletion, including KCs) (Lanaya et al, 2014) (Fig 1A). Poly-I:C or tamoxifen were injected to induce the expression of the cre-recombinase in the Mx-cre or to activate the creER-recombinase in Alb-cre strains, respectively, along with control mice. Liver fibrosis was induced by bi-weekly CCl_4_ injections, as shown in the work-flow and treatment protocol diagram (Fig 1B), and all mice were euthanized after 8 wk of treatment for detailed analyses. Evaluation of deletion of *c-jun* using whole livers at euthanasia showed extensive *c-jun* deletion in the different groups of mice (Fig S2B). We further purified hepatocytes and KCs from the respective cohorts of mice, which again showed specific and significant deletion only in the hepatocytes from the c-jun^f/f^;Alb-cre mice or in both the hepatocytes and KCs in livers from the c-jun^f/f^;Mx-cre mice (Fig S2C).

c-Jun expression was significantly reduced in livers of c-jun^f/f^;Mx-cre and c-jun^f/f^;Alb-cre mice at euthanasia (Fig 1C), highlighting the impact of its deletion in hepatocytes that account for more than 80% of the cellular population in the liver, as previously reported (Behrens et al, 2002). It is noteworthy that Alb-cre–mediated *c-jun* deletion resulted in slightly higher residual levels of c-Jun in the livers than that of Mx-cre–mediated deletion (∼80% and 97% decrease in c-Jun expression in livers of c-jun^f/f^;Alb-cre and c-jun^f/f^;Mx-cre, respectively), likely reflecting the expansive deletion beyond hepatocytes in the latter case.

Sirius red staining revealed a significant increase in fibrosis in the c-jun^f/f^;Alb-cre mice (% Sirius red staining of CCl_4_-treated livers: without or with Alb-cre - 6.003 ± 1.958 versus 18.614 ± 10.407; *P* = 0.009) (Fig 1D, left). However and strikingly, *c-jun* deletion in the c-jun^f/f^;Mx-cre mice led to a consistent decrease in fibrosis (% Sirius red staining of CCl_4_-treated livers: without or with Mx-cre – 4.698 ± 0.709 versus 4.018 ± 0.476; *P* = 0.033) (Fig 1D, right), indicating that *c-jun* deletion in hepatocytes alone or in combination with KCs result in differential outcomes. A similar trend was observed with alanine aminotransferase (ALT) and aspartate aminotransferase (AST) levels (Fig 1E). These results together suggest a causal role for c-Jun in regulating liver fibrosis, likely through interplay between the hepatocytes and KCs.

### Differential effects on cytokine gene expression upon *c-jun* deletion in hepatocytes alone or in combination with KCs

**Figure 2.**
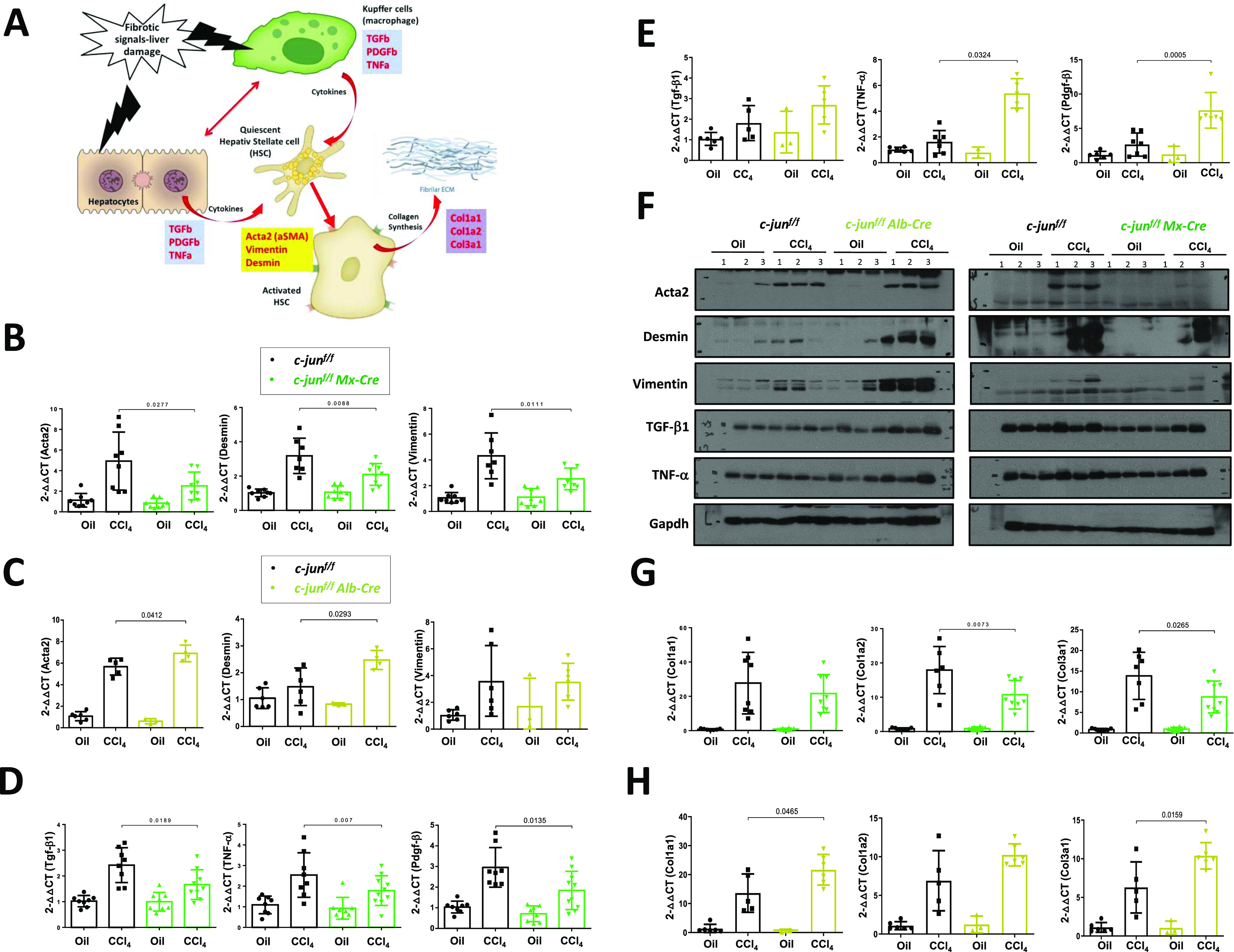
*c-jun* deletion in hepatocytes alone or together with Kupffer cells affects cytokine gene expression differentially. **(A)** Schematic of genes analyzed by quantitative real-time (qRT)-PCR to determine the role of c-Jun in the liver. The three groups of genes analyzed include cytokines produced by hepatocytes and Kupffer cells, hepatic stellate cell activation markers and fibrogenic procollagen genes. **(B, C)** qRT-PCR analysis was performed using livers from c-jun^f/f^ and c-jun^f/f;^Mx-cre mice (B) or c-jun^f/f^ and c-jun^f/f;^Alb-cre mice (C), to determine the expression of *Acta2*, *Desmin* and *Vimentin*, which were normalized against *Gapdh*. Experiments were performed in duplicates, and data represent mean ± SD. Each dot represents a liver from each mouse. n = c-jun^f/f^: oil – 6; CCl_4_ – 5/6; c-jun^f/f^;Alb-cre: oil – 2/3; CCl_4_ – 4-6; c-jun^f/f^: oil – 8; CCl_4_ – 7/8; c-jun^f/f^;Mx-cre: oil – 8; CCl_4_ – 7/8. **(D, E)** Similar qRT-PCR analysis was performed to determine the expression of *Tgf-*β*1*, *Tnf-*α and *Pdgf-*β, which were normalized against *Gapdh*. Experiments were performed in duplicates, and data represent mean ± SD. Each dot represents a liver from each mouse. n = c-jun^f/f^: oil – 6; CCl_4_ – 5-7; c-jun^f/f^;Alb-cre: oil – 2/3; CCl_4_ – 5/6; c-jun^f/f^: oil – 8; CCl_4_ – 8; c-jun^f/f^;Mx-cre: oil – 8; CCl_4_ – 9/10. **(F)** Whole liver lysates from the indicated groups of mice were used for immunoblotting analysis with the indicated antibodies. The Gapdh blot was performed once for all lysates, and the blot shown here is the same as shown in Figs 1C and S1D left panel, as the same set of lysates was used in these figures. **(G, H)** qRT-PCR analysis was performed as described above to determine the expression of *Col1*α*1*, *Col1*α*2* and *Col3*α*1*, which were normalized against *Gapdh*. Experiments were performed in duplicates, and data represent mean ± SD. Each dot represents a liver from each mouse. n = c-jun^f/f^: oil – 6; CCl_4_ – 5; c-jun^f/f^;Alb-cre: oil – 2/3; CCl_4_ – 6; c-jun^f/f^: oil – 8; CCl_4_ – 6-8; c-jun^f/f^;Mx-cre: oil – 8; CCl_4_ – 8/9. Statistics was performed by unpaired *t* test for all qRT-PCR data. Source data are available for this figure.

To understand the molecular basis for the observed differential effects of *c-jun* deletion in hepatocytes alone or in combination with KCs, we analyzed the expression of selected genes from three groups corresponding to various processes that coordinately regulate liver fibrosis: (i) *Acta2* (α*-SMA*), *Desmin* and *Vimentin*, whose elevated expression reflects the activation status of the HSCs; (ii) *Tgf-*β*1*, *Tnf-*α, and *Pdgf-*β, the inflammatory cytokines that are produced by hepatocytes and KCs to induce the fibrotic response; and finally, (iii) *Col1*α*1*, *Col1*α*2*, and *Col3*α*1*, whose expression is induced for collagen synthesis and deposition by the activated HSCs (Fig 2A). Quantitative real-time (qRT) PCR analysis revealed that HSC activation, as measured by *Acta2*, *Desmin*, and *Vimentin* expression, was compromised in c-jun^f/f^;Mx-cre mice, corroborating with the observed reduction in fibrosis (relative expression of *Acta2* in livers of CCl_4_-treated mice: without or with Mx-cre - 4.931 ± 2.816 versus 2.523 ± 1.341, *P* = 0.0277; *Desmin* - 3.182 ± 1.028 versus 2.096 ± 0.634, *P* = 0.0088; *Vimentin* - 4.319 ± 1.780 versus 2.528 ± 0.840, *P* = 0.0111) (Fig 2B). On the contrary, and consistent with the observed increased fibrosis, there was a tendency for enhanced HSC activation in the c-jun^f/f^;Alb-cre mice (relative expression of *Acta2* in livers of CCl_4_-treated mice: without or with Alb-cre - 5.679 ± 0.7799 versus 6.912 ± 0.7711, *P* = 0.0412; *Desmin* - 1.479 ± 0.7008 versus 2.476 ± 0.3532, *P* = 0.0293) (Fig 2C).

We, therefore, evaluated if the induction of the fibrotic response contributed to the deregulated effects observed on HSCs. Analyses of a few cytokines gene expression revealed a defect in several of them, including *Tgf-*β*1*, *Tnf-*α and *Pdgf-*β in c-jun^f/f^;Mx-cre mice (relative expression of *Tgfb1* in livers of CCl_4_-treated mice: without or with Mx-cre - 2.422 ± 0.6773 versus 1.663 ± 0.5785, *P* = 0.0189; *Tnf-*α - 2.539 ± 1.080 versus 1.786 ± 0.7205, *P* = 0.007; *Pdgf-*β - 2.958 ± 0.9613 versus 1.843 ± 0.9280, *P* = 0.0135) (Fig 2D), but an opposite effect was generally noted in c-jun^f/f^;Alb-cre mice (relative expression of *Tnf-*α in livers of CCl_4_-treated mice: without or with Alb-cre - 1.620 ± 0.8758 versus 5.385 ± 1.143, *P* = 0.0324; *Pdgf-*β - 2.640 ± 1.686 versus 7.629 ± 2.604, *P* = 0.0005) (Fig 2E), suggesting that the differential effects observed on HSC activation may stem from the alterations in the initial process of fibrosis induction. Consistent with the qRT-PCR data, expression of Acta2, Desmin, Vimentin, TNF-α and TGF-β1 was generally higher in the livers of c-jun^f/f^;Alb-cre mice compared with the c-jun^f/f^ controls, but lower in the c-jun^f/f^;Mx-cre mice (Fig 2F).

Furthermore and consistent with the altered HSC activation, production of collagen synthesis was also compromised in the livers of the c-jun^f/f^;Mx-cre mice (relative expression of *Col1a1* in livers of CCl_4_-treated mice: without or with Mx-cre - 27.71 ± 17.93 versus 21.64 ± 11.07, *P* = 0.6616; *Col1a2* - 17.91 ± 6.877 versus 10.74 ± 4.176, *P* = 0.0073; *Col3a1* - 13.87 ± 5.735 versus 8.751 ± 3.871, *P* = 0.0265), but generally increased in the c-jun^f/f^;Alb-cre mice (relative expression of *Col1a1* in livers of CCl_4_-treated mice: without or with Alb-cre - 13.62 ± 6.634 versus 21.66 ± 5.286, *P* = 0.0465; *Col1a2* - 6.897 ± 3.891 versus 10.24 ± 1.427, *P* = 0.0710; *Col3a1* - 6.285 ± 3.303 versus 10.38 ± 1.713, *P* = 0.0159) (Fig 2G and H). Collectively, these data indicate that the reduced expression of the cytokine genes in c-jun^f/f^;Mx-cre mice and vice versa in c-jun^f/f^;Alb-cre mice is the likely cause of the differential effects observed on HSC activation and fibrosis after *c-jun* deletion, either in hepatocytes alone or together with KCs.

### Non-cell autonomous effects are causal to increased fibrosis upon hepatocyte-specific *c-jun* deletion

**Figure 3.**
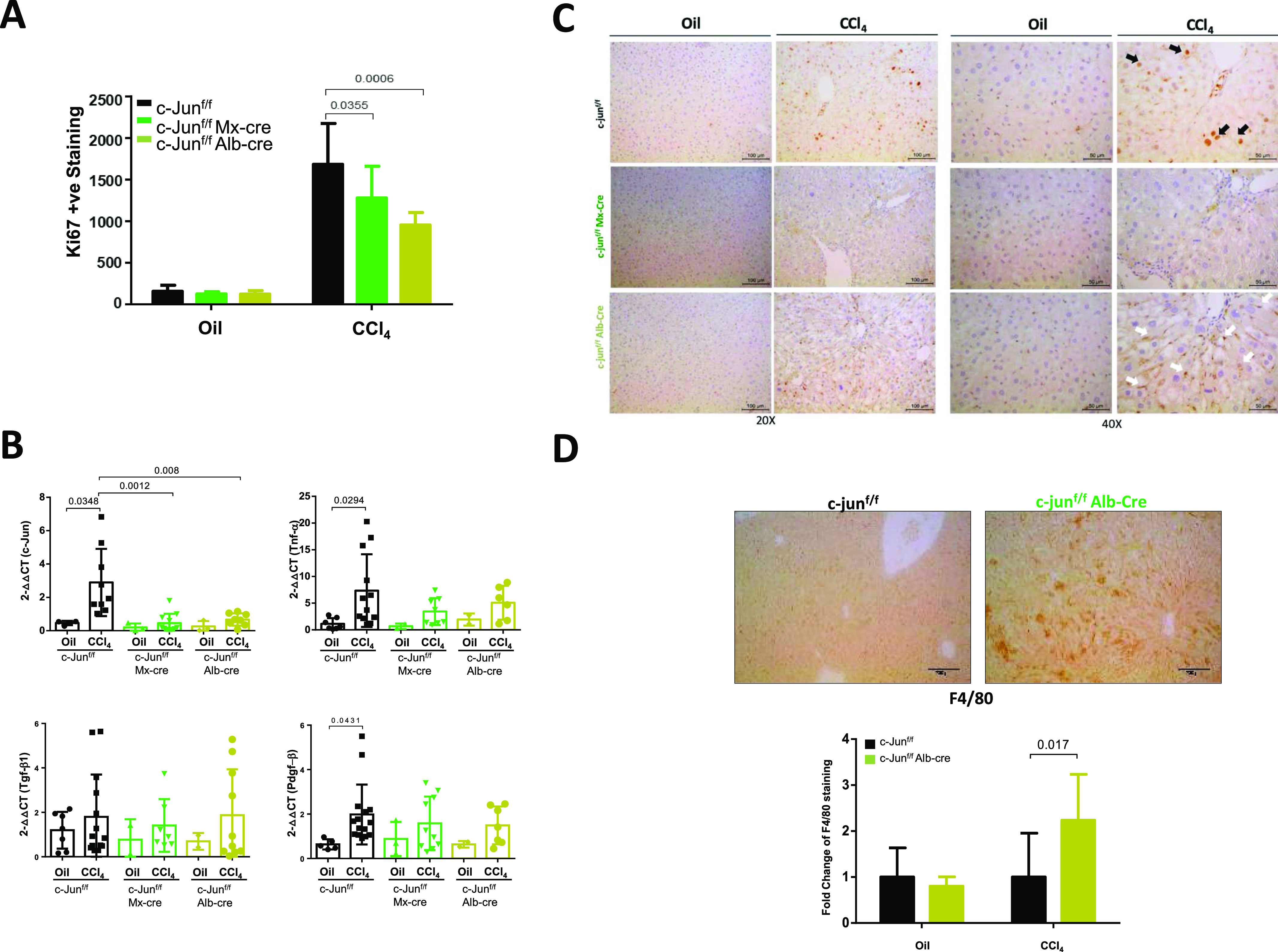
Increase in Kupffer cells in livers of mice with hepatocyte-specific *c-jun* deletion. **(A)** The number of proliferating cells was determined on the liver sections by Ki67 staining. Data represent mean ± SD. For Ki67 staining, n = c-jun^f/f^: oil – 9; CCl_4_ – 14; c-jun^f/f^;Alb-cre: oil – 5; CCl_4_ – 6; c-jun^f/f^;Mx-cre: oil – 4; CCl_4_ – 6. Statistics was performed by unpaired by *t* test. **(B)** Purified hepatocytes from c-jun^f/f^, c-jun^f/f;^Mx-cre and c-jun^f/f^;Alb-cre mice were used to determine the expression of *c-jun* and indicated cytokine genes, as described. Data represent mean ± SD. n = c-jun^f/f^: oil – 5-7; CCl_4_ – 9-15; c-jun^f/f^;Alb-cre: oil – 2/3; CCl_4_ – 7-10; c-jun^f/f^;Mx-cre: oil – 2/3; CCl_4_ – 6-10. Statistics was performed by unpaired *t* test. **(C, D)** Expression of c-Jun was determined by immunohistochemical staining of liver tissues from the indicated mouse strains after 8 wk of CCl_4_ or oil injection. Representative images are shown. Black arrows indicate the presence of c-Jun positive hepatocytes (reddish brown). **(C)** White arrows indicate c-Jun positive macrophage-like cells (C). Numbers of F4/80 positive macrophages were quantified (bottom panel) after immunohistochemical staining. **(D)** Representative pictures are shown (top panel) (D). Data represent mean + SD. c-jun^f/f^: oil – 10; CCl_4_ – 14; c-jun^f/f^;Alb-cre: oil – 2; CCl_4_ – 6. Statistics was performed by unpaired *t* test.

To understand the observed differential effects, we first evaluated the levels of proliferation (by Ki67 staining) and cell death (by TUNEL staining) in the livers of the CCl_4_-treated c-jun^f/f^;Alb-cre and c-jun^f/f^;Mx-cre mice. Expectedly, the numbers of Ki67^+^ cells were reduced in both the c-jun^f/f^;Alb-cre mice and c-jun^f/f^;Mx-cre mice (Fig 3A). This observation is consistent with previous findings that c-Jun is required for the proliferation of hepatocytes upon liver injury (Behrens et al, 2002). There were also no differences noted in cell death at the point of euthanasia in the c-jun^f/f^;Mx-cre mice (Fig S2D). We, therefore, purified hepatocytes from both groups of mice and evaluated the expression of several cytokine genes which were induced by CCl_4_ treatment. Interestingly, hepatocytes from both groups of mice in which *c-jun* was deleted (Fig S2C) had a significant and similar reduction in the induction of these cytokine genes (Fig 3B), suggesting that the observed differential effects on fibrosis may not be attributable to the differential effects of *c-jun* deletion in hepatocytes in these two cases.

We, therefore, hypothesized that there may be compensatory or counteracting mechanisms in place to mitigate the lack of hepatocyte function in the c-jun^f/f^;Alb-cre cohorts, leading to the observed enhanced fibrosis. Although immunohistochemical analysis showed a significant reduction of c-Jun expression in hepatocytes in both groups of mice after CCl_4_ treatment, we noted that there was an increase in the number of macrophage-like cells infiltrating the liver in the c-jun^f/f^;Alb-cre mice with significant levels of c-Jun expression (Fig 3C). To confirm the identity of the cells, we stained the sections with F4/80 to detect macrophages, which were enriched in the livers from the c-jun^f/f^;Alb-cre mice (Fig 3D), suggesting that elevated levels of KCs and infiltrating macrophages in the absence of *c-jun* may be causal to the observed enhanced fibrosis in this group of mice.

### Monocyte-specific *c-jun* deletion results in reduced fibrosis

**Figure S3.**
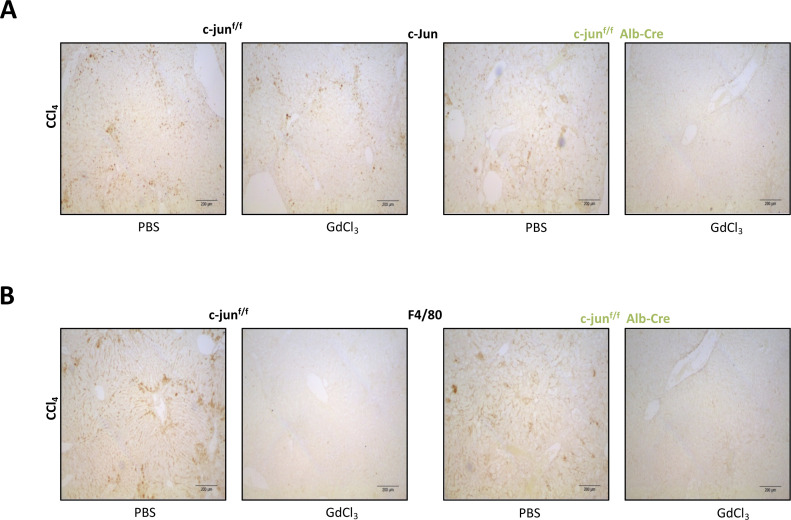
Immunohistochemcial analysis of liver upon *c-jun* deletion and depletion of macrophages. **(A, B)** c-jun^f/f^ or c-jun^f/f^;Alb-cre mice were treated once a week for 8 wk with PBS or gadolinium chloride (GdCl_3_) to deplete the macrophages, and concomitantly with oil or CCl_4_ treatment to induce fibrosis, and their livers were harvested and used for immunostaining to determine the expression of c-Jun (A) or macrophages by staining with anti-F4/80 antibody (B). Representative pictures are shown.

**Figure 4.**
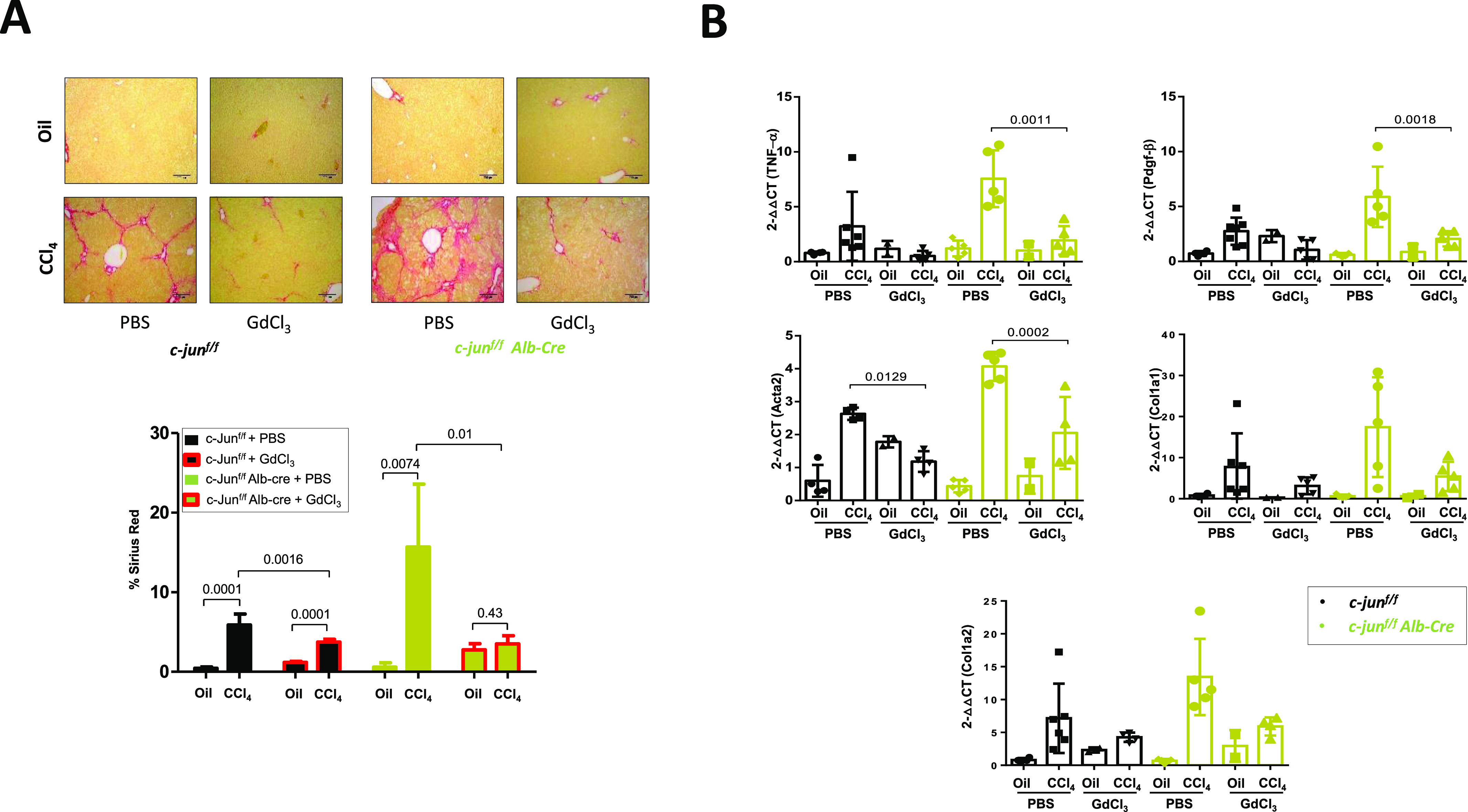
Depletion of macrophages reverses fibrosis in mice with hepatocyte-specific *c-jun* deletion. **(A, B)** c-jun^f/f^ or c-jun^f/f^;Alb-cre mice were treated with gadolinium chloride (GdCl_3_) or PBS once a week for 8 wk to deplete the macrophages, concomitant with oil or CCl_4_ treatment. **(A, B)** The extent of fibrosis (A), and the expression of the indicated genes from whole livers (B) were then evaluated as described. Experiments were performed in duplicates, and data represent mean ± SD. **(B)** Each dot represents a liver from each mouse (B). n = c-jun^f/f^: oil/PBS – 4; CCl_4_/PBS – 4-7; oil/GdCl_3_ – 2; CCl_4_/GdCl_3_ – 4-6; c-jun^f/f^;Alb-cre: oil/PBS – 5; CCl_4_/PBS – 5; oil/GdCl_3_ – 2; CCl_4_/GdCl_3_ – 4/5. Statistics was performed by two-way ANOVA.

To investigate the causal role of macrophages to the observed increased fibrosis, we evaluated the effects of depleting macrophages from c-jun^f/f^;Alb-cre mice. To this end, c-jun^f/f^;Alb-cre mice were treated gadolinium chloride (GdCl_3_), which has been shown to deplete macrophages in vivo (Zhu et al, 2018). GdCl_3_ treatment led to a significant reduction in c-Jun expression in livers of CCl_4_-treated c-jun^f/f^;Alb-cre mice (Fig S3A), and a concomitant decrease in F4/80 expressing cells (Fig S3B). Analysis of fibrosis by Sirius red staining also showed a reversal and marked reduction of fibrosis in CCl_4_-treated c-jun^f/f^;Alb-cre mice upon GdCl_3_ treatment (% Sirius red staining of CCl_4_-treated livers: without or with Alb-cre: for PBS - 5.875 ± 1.384 versus GdCl_3_ - 3.734 ± 0.341, *P* = 0.0016; and PBS – 15.698 ± 7.898 versus GdCl_3_ - 3.502 ± 1.029, *P* = 0.01) (Fig 4A). Further analysis of expression of multiple genes regulating HSC activation, cytokine production, and collagen synthesis revealed a concomitant reversal of all parameters in GdCl_3_ treated c-jun^f/f^;Alb-cre mice (Fig 4B), implying a compensatory contributory role for KCs to the enhanced liver fibrosis observed in the c-jun^f/f^;Alb-cre mice.

**Figure 5.**
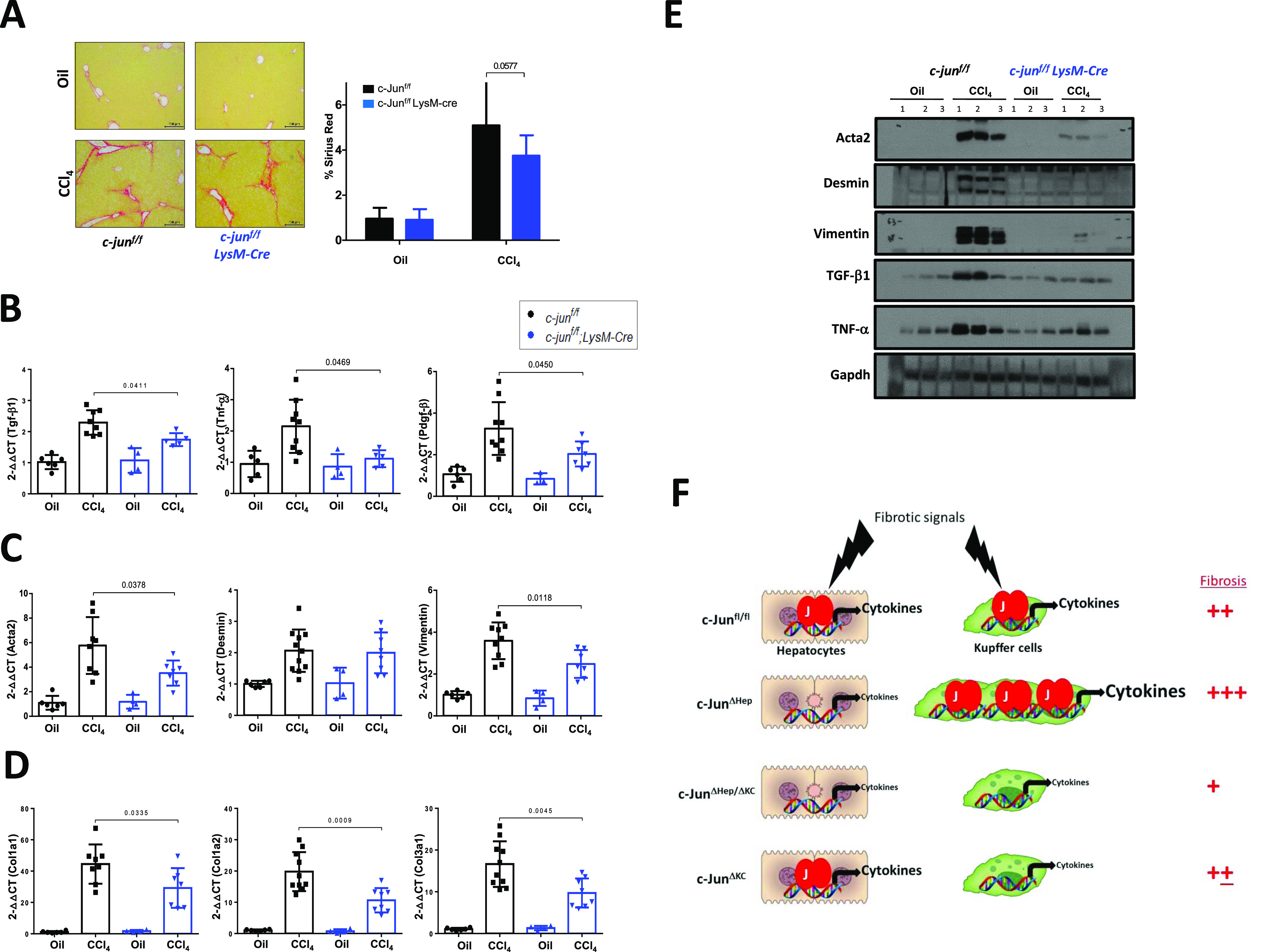
c-Jun is a positive regulator of fibrosis in Kupffer cells (KCs). **(A)** Extent of fibrosis was determined by Sirius red staining (left panels), and quantified (right panels) using livers from c-jun^f/f^ and c-jun^f/f^;LysM-cre mice (A). Representative pictures are shown. Data represent mean ± SD. n = c-jun^f/f^: oil – 5; CCl_4_ – 7; c-jun^f/f^;LysM-cre: oil – 4; CCl_4_ – 7. Statistics was performed by unpaired *t* test. **(B, C, D, E)** Quantitative real-time-PCR analysis was performed using livers from c-jun^f/f^ and c-jun^f/f^;LysM-cre mice, to determine the expression of the indicated genes (B, C, D). Experiments were performed in duplicates, and data represent mean ± SD. Each dot represents a liver from each mouse. n = c-jun^f/f^: oil – 5/6; CCl_4_ – 8-11; c-jun^f/f^;LysM-cre: oil – 3/4; CCl_4_ – 5-8. Statistics was performed by unpaired *t* test. **(E)** Whole liver lysates from the indicated groups of mice were used for immunoblotting analysis with the indicated antibodies as described above (E). **(F)** Compensation by macrophages for the lack of c-Jun function specifically in hepatocytes. Fibrotic signals activate c-Jun in hepatocytes and KCs, leading to production of cytokines and thus fibrosis (extent arbitrarily indicated by ++). In the absence of c-Jun in hepatocytes only, the KCs over-compensate for the deficiency, which results in overall elevated fibrosis (+++). However, in the absence of c-Jun in hepatocytes and KCs, or KCs alone, cytokine production is diminished, and results in reduced fibrosis (+ or +±). Source data are available for this figure.

We, therefore, next evaluated if *c-jun* deletion in F4/80^+^ monocytic lineage cells alone would also lead to reduced fibrosis. To this end, *c-jun* was deleted using the LysM-cre mice (Fig S2B), which led to a consistent decrease in CCl_4_-mediated fibrosis (% Sirius red staining: without or with LysM-cre - 5.091 ± 2.143 versus 3.747 ± 0.908, *P* = 0.0577) (Fig 5A). Importantly, the cytokine genes expression was significantly impaired in the c-jun^f/f^;LysM-cre mice (relative expression of *Tgfb1* in livers of CCl_4_-treated mice: without or with LysM-cre – 2.293 ± 0.3946 versus 1.742 ± 0.2081, *P* = 0.0411; *Tnf-*α - 2.150 ± 0.8507 versus 1.114 ± 0.2720, *P* = 0.0469; *Pdgf-*β – 3.259 ± 1.267 versus 2.037 ± 0.5995, *P* = 0.045) (Fig 5B). Consistently, HSC activation and collagen synthesis were also compromised by deletion of *c-jun* in the monocytic cells (relative expression of *Acta2* in livers of CCl_4_-treated mice: without or with LysM-cre – 5.771 ± 2.320 versus 3.521 ± 1.026, *P* = 0.0378; *Desmin* – 2.065 ± 0.6768 versus 1.997 ± 0.6518, *P* = 0.8297; *Vimentin* – 3.590 ± 0.8778 versus 2.483 ± 0.6671, *P* = 0.0118; *Col1*α*1* – 44.49 ± 12.51 versus 29.24 ± 12.64, *P* = 0.0335; *Col1*α*2* – 19.81 ± 6.227 versus 10.64 ± 3.899, *P* = 0.0009; *Col3*α*1* – 16.64 ± 5.464 versus 9.720 ± 3.472, *P* = 0.0045) (Fig 5C and D). As noted with the livers of the c-jun^f/f^;Mx-cre mice, the expression of Acta2, Desmin, Vimentin, TNF-α and TGF-β1 was significantly reduced in the livers of c-jun^f/f^;LysM-cre mice compared to the c-jun^f/f^ controls (Fig 5E). Collectively, these data indicate that c-Jun is required for the efficient expression of cytokine genes in hepatocytes as well as the F4/80^+^ macrophages, both of which contribute to liver fibrosis. However, absence of c-Jun in hepatocytes alone leads to a compensatory increase in the functions of KCs, which therefore lead to enhanced fibrosis in the case of the c-jun^f/f^;Alb-cre mice.

### *c-jun* deletion does not affect fibrosis resolution

**Figure 6.**
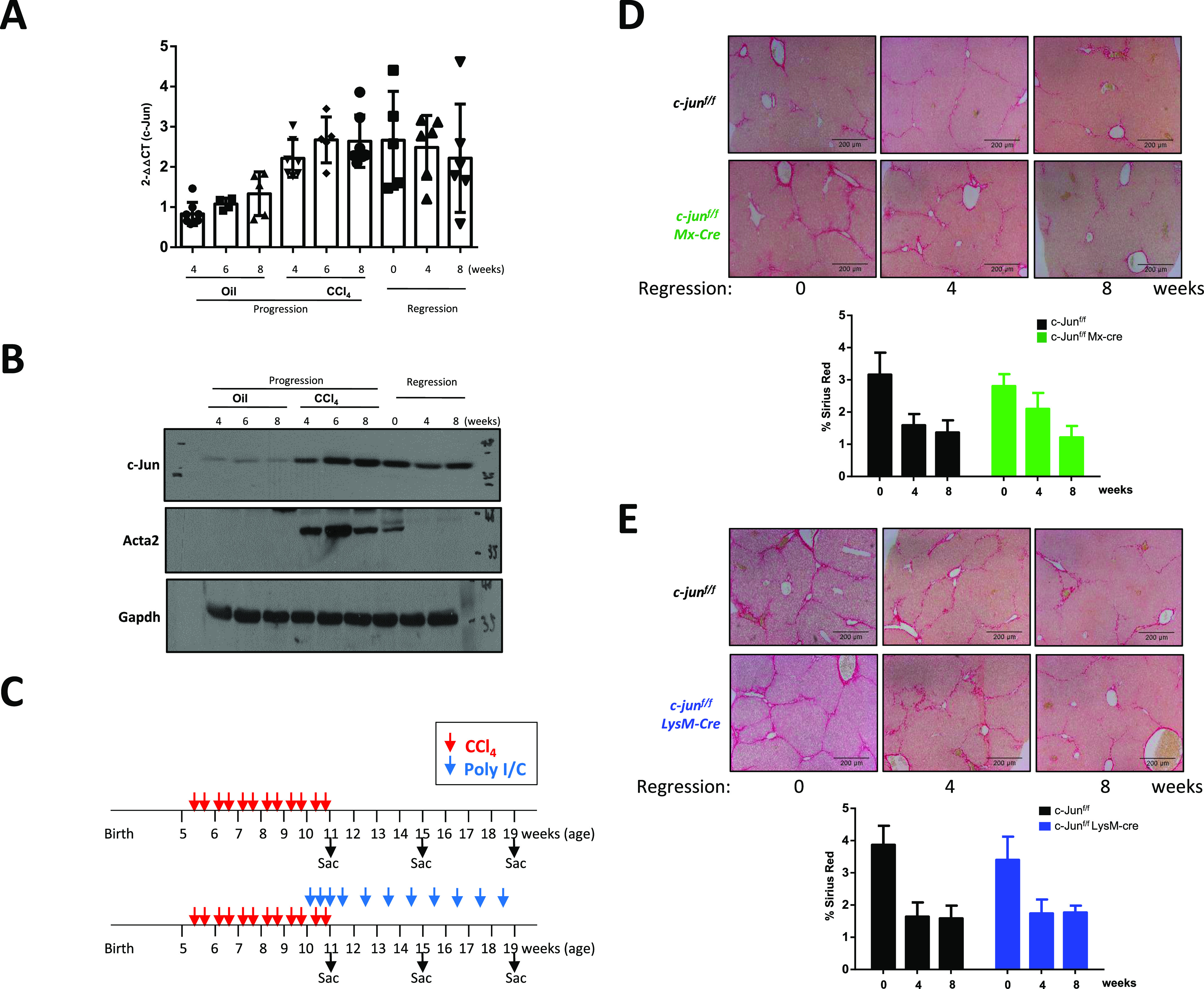
*c-jun* deletion in Kupffer cells alone or together with hepatocytes does not affect fibrosis resolution. **(A, B)** Expression of *c-jun* was determined by quantitative real-time-PCR (A) and by immunoblotting (B) using whole livers from mice treated with oil or CCl_4_-tretment (progression), and for 8 wk posttreatment (regression). Experiments were performed in duplicates and data represent mean ± SD. Each dot represents a liver from an individual mouse. n = c-jun^f/f^: oil: 4 wk – 8; 6 wk – 4; 8 wk – 5; c-jun^f/f^: CCl_4_ : 4 wk – 7; 6 wk – 5; 8 wk – 7; c-jun^f/f^ Regression: 0 wk – 5; 4 wk – 6; 8 wk – 6. Statistics was performed by unpaired *t* test. **(C)** Schematic of fibrosis regression studies. Mice were either treated with oil or CCl_4_ twice per week for 6 wk to induce fibrosis. Poly I:C was injected as indicated to induce the expression of Cre in Mx-cre, from the last week of CCl_4_ injection till the end of the experimental period of 8 wk post-CCl_4_ treatment. Mice were harvested at either 0, 4, or 8 wk from the cessation of CCl_4_ treatment. **(D, E)** Extent of liver fibrosis and damage over 8 wk post-CCl_4_ treatment was determined by Sirius red staining (top panels), and representative pictures are shown (10× magnification). Quantification of fibrosis is based on analyzing 20 randomly chosen fields from four individual liver lobes per mouse (lower panels). Data represent mean + SD. n = c-jun^f/f^: 0 wk – 8; 4 wk – 8; 8 wk - 7; c-jun^f/f^;Mx-cre: 0 wk – 8; 4 wk – 7; 8 wk – 8; c-jun^f/f^: 0 wk – 9; 4 wk – 8; 8 wk – 10; c-jun^f/f^;LysM-cre: 0 wk – 8; 4 wk – 6; 8 wk – 6. Statistics was performed by two-way ANOVA. Source data are available for this figure.

**Figure S4.**
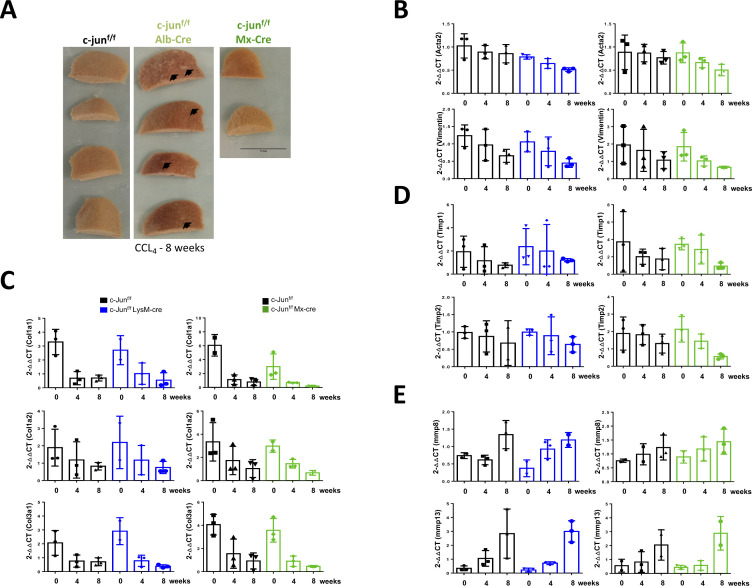
Molecular analysis of fibrosis resolution in the absence of c-Jun. **(A)** Macroscopic view of livers from the indicated groups of mice is shown after 8 wk of CCl_4_ treatment. White arrows show macroscopically visible nodules in c-jun^f/f^;Alb-cre mice which were absent in c-jun^f/f^ and c-jun^f/f^;Mx-cre mice. **(B, C, D, E)** Quantitative real-time-PCR analysis was performed using RNA from whole livers from c-jun^f/f^ and c-jun^f/f^;Mx-cre mice or c-jun^f/f^ and c-jun^f/f^;LysM-cre mice, to determine the expression of *Acta2* and *Vimentin* (B), *Col1*α*1*, *Col1*α*2* and *Col3*α*1* (C), *Timp1* and *Timp2* (D), and *mmp8* and *mmp13* (E), which were normalized against *Gapdh*. Experiments were performed in duplicates, and data represent mean ± SD. Each dot represents a liver from each mouse. n = c-jun^f/f^: 0 – 2/3; 4 – 3; 8 – 2/3; c-jun^f/f^;LysM-cre: 0 – 2/3; 4 – 3; 8 – 2/3; c-jun^f/f^: 0 – 2/3; 4 – 2/3; 8 – 2/3; c-jun^f/f^;Mx-cre: 0 – 2/3; 4 – 3; 8 – 2/3.

Given the significant role of c-Jun in mediating liver fibrosis through both the hepatocytes and KCs, we further evaluated if it is also involved in the resolution phase, after removal of the fibrotic stimuli. Initial analysis of *c-jun* expression, which is induced by CCl_4_ treatment, indicated that the expression was maintained over 8-wk posttreatment (Fig 6A). Similar results were obtained by immunoblot analysis despite the almost complete decrease in Acta2 expression, reflecting the resolution of fibrosis (Fig 6B). Hence, to evaluate if there is a causal role for c-Jun in the resolution process, fibrosis was induced by CCl_4_ treatment for 6 wk, and the livers were analyzed posttreatment. We used the c-jun^f/f^;Mx-cre and c-jun^f/f^;LysM-cre mice for analysis, as the c-jun^f/f^;Alb-cre mice already started developing nodules on the livers after 8 wk of CCl_4_ treatment (Fig S4A), and hence, were not included in this study. c-jun^f/f^;Mx-cre mice were injected with Poly-I:C 1 wk before the end of the CCl_4_ treatment to delete *c-jun*, which continued till the end of the experiment (Figs 6C and S2E), and livers were harvested at 4 and 8 wk post-CCl_4_ treatment and analyzed. As shown in Fig 6D, deletion of *c-jun* did not markedly affect the fibrosis resolution process in the c-jun^f/f^;Mx-cre mice (% Sirius red staining of CCl_4_-treated livers: without or with Mx-cre at the following time points [weeks] – 0: 3.160 ± 0.684 versus 2.805 ± 0.374; *P* = 0.6294; 4: 1.588 ± 0.353 versus 2.098 ± 0.496; *P* = 0.2787; 8: 1.363 ± 0.379 versus 1.215 ± 0.353; *P* = 0.9880). Consistently, whereas the molecular markers associated with fibrosis decreased over time, no significant differences were noted because of the absence of c-Jun, as shown for *Acta2* and *Vimentin* (relative expression of *Acta2* in livers of CCl_4_-treated regressed mice: without or with Mx-cre at the following time points [weeks] – 0: 0.8850 ± 0.3717 versus 0.8735 ± 0.2132; *P* = 0.9999; 4: 0.8686 ± 0.1878 versus 0.6636 ± 0.1108; *P* = 0.8310; 8: 0.7665 ± 0.1349 versus 0.5015 ± 0.1239; *P* = 0.6452; *Vimentin* – 0: 1.946 ± 1.079 versus 1.844 ± 0.8240; *P* = 0.9999; 4: 1.632 ± 1.210 versus 1.027 ± 0.2873; *P* = 0.9243; 8: 1.071 ± 0.4948 versus 0.6627 ± 0.06152; *P* = 0.9851) (Fig S4B), and *Col1*α*1*, *Col1*α*2*, and *Col3*α*1* (relative expression of *Col1a1* in livers of CCl_4_-treated regressed mice: without or with Mx-cre at the following time points [weeks] – 0: 6.066 ± 1.557 versus 2.998 ± 1.823; *P* = 0.049; 4: 1.127 ± 0.6879 versus 0.6947 ± 0.06344; *P* = 0.9931; 8: 0.7872 ± 0.5212 versus 0.2041 ± 0.1185; *P* = 0.9742; *Col1a2* – 0: 3.342 ± 1.655 versus 2.973 ± 0.5303; *P* = 0.9983; 4: 1.733 ± 1.222 versus 1.456 ± 0.3733; *P* = 0.9992; 8: 1.037 ± 0.7700 versus 0.6706 ± 0.2067; *P* = 0.9983; *Col3a1* – 0: 4.056 ± 0.8880 versus 3.568 ± 1.020; *P* = 0.9751; 4: 1.550 ± 1.257 versus 0.9002 ± 0.4455; *P* = 0.9211; 8: 0.9227 ± 0.6937 versus 0.4520 ± 0.08341; *P* = 0.9787) (Fig S4C). Moreover, the expression of the tissue inhibitor of metalloproteinases (TIMPs) 1 and 2 which are down-regulated during liver fibrosis resolution, as well as the matrix metellopeptidases (MMPs) 8 and 13 which are induced during fibrosis resolution (Giannandrea & Parks, 2014) were not significantly altered because of the absence of c-Jun (relative expression of *Timp1* in livers of CCl_4_-treated regressed mice: without or with Mx-cre at the following time points [weeks] –– 0: 3.722 ± 3.475 versus 3.444 ± 0.6431; *P* = 0.9999; 4: 2.015 ± 0.8716 versus 2.842 ± 1.661; *P* = 0.9898; 8: 1.750 ± 1.226 versus 0.9351 ± 0.3654; *P* = 0.9904; *Timp2* – 0: 1.884 ± 0.9538 versus 2.130 ± 0.7394; *P* = 0.9957; 4: 1.814 ± 0.5711 versus 1.443 ± 0.4122; *P* = 0.9729; 8: 1.308 ± 0.5455 versus 0.5661 ± 0.1434; *P* = 0.6839; *mmp8* – 0: 0.7559 ± 0.05902 versus 0.8920 ± 0.2225; *P* = 0.9991; 4: 0.9880 ± 0.3799 versus 1.177 ± 0.4334; *P* = 0.9933; 8: 1.229 ± 0.4459 versus 1.438 ± 0.499; *P* = 0.9829; *mmp13* – 0: 0.5594 ± 0.4620 versus 0.4415 ± 0.1429; *P* = 0.9999; 4: 0.8231 ± 0.7412 versus 0.5764 ± 0.3448; *P* = 0.9972; 8: 2.048 ± 1.091 versus 2.889 ± 1.211; *P* = 0.8158) (Fig S4D and E). Similar results were obtained using c-jun^f/f^;LysM-cre mice for fibrosis resolution (% Sirius red staining of CCl_4_-treated livers: without or with LysM-cre at the following time points [weeks] – 0: 3.869 ± 0.589 versus 3.401 ± 0.722; *P* = 0.4128; 4: 1.640 ± 0.443 versus 1.741 ± 0.433; *P* = 0.9990; 8: 1.584 ± 0.399 versus 1.768 ± 0.215; *P* = 0.9820), and the expression of the molecular markers (relative expression of *Acta2* in livers of CCl_4_-treated regressed mice: without or with LysM-cre at the following time points [weeks] – 0: 1.024 ± 0.2616 versus 0.785 ± 0.04748; *P* = 0.4489; 4: 0.8891 ± 0.1436 versus 0.6433 ± 0.1056; *P* = 0.4197; 8: 0.8557 ± 0.1970 versus 0.5133 ± 0.03898; *P* = 0.1421; *Vimentin* – 0: 1.235 ± 0.3104 versus 1.058 ± 0.2877; *P* = 0.9803; 4: 0.9683 ± 0.4521 versus 0.7856 ± 0.4128; *P* = 0.9777; 8: 0.6567 ± 0.1874 versus 0.4483 ± 0.1257; *P* = 0.9613; *Col1a1* – 0: 3.293 ± 0.9085 versus 2.706 ± 1.046; *P* = 0.9244; 4: 0.6908 ± 0.4724 versus 1.017 ± 0.7698; *P* = 0.9896; 8: 0.7012 ± 0.2114 versus 0.5485 ± 0.5340; *P* = 0.9997; *Col1a2* – 0: 1.895 ± 1.059 versus 2.194 ± 1.508; *P* = 0.9988; 4: 1.185 ± 1.044 versus 1.175 ± 0.8429; *P* = 0.9999; 8: 0.8234 ± 0.2167 versus 0.7506 ± 0.3485; *P* = 0.9999; *Col3a1* – 0: 2.069 ± 0.8903 versus 2.912 ± 0.9697; *P* = 0.5828; 4: 0.7596 ± 0.4374 versus 0.7815 ± 0.4024; *P* = 0.9999; 8: 0.7119 ± 0.2856 versus 0.3862 ± 0.1129; *P* = 0.9760; *Timp1* – 0: 1.934 ± 1.347 versus 2.371 ± 1.564; *P* = 0.9984; 4: 1.159 ± 1.209 versus 1.995 ± 2.295; *P* = 0.9704; 8: 0.7730 ± 0.2090 versus 1.209 ± 0.1380; *P* = 0.9984; *Timp2* – 0: 0.9842 ± 0.1702 versus 0.9948 ± 0.09253; *P* = 0.9999; 4: 0.8715 ± 0.4498 versus 0.8904 ± 0.5435; *P* = 0.9999; 8: 0.6796 ± 0.6441 versus 0.6415 ± 0.2215; *P* = 0.9999; *mmp8* – 0: 0.7377 ± 0.08479 versus 0.3740 ± 0.2410; *P* = 0.7365; 4: 0.6134 ± 0.1370 versus 0.9259 ± 0.2685; *P* = 0.6974; 8: 1.343 ± 0.4060 versus 1.190 ± 0.2151; *P* = 0.9848; *mmp13* – 0: 0.3456 ± 0.1746 versus 0.2246 ± 0.1359; *P* = 0.9999; 4: 1.057 ± 0.5640 versus 0.7145 ± 0.1054; *P* = 0.9948; 8: 2.840 ± 1.767 versus 3.000 ± 0.7627; *P* = 0.9999) (Figs 6E and 4B–E). These results together indicate that c-Jun is not required for the fibrosis resolution process.

## Discussion

We have in this study investigated the interplay between cell types in orchestrating the liver fibrotic process, using c-Jun deficiency in hepatocytes and macrophages as a model. c-Jun, being a critical transcription factor implicated in the biology of hepatocytes (Hilberg et al, 1993; Behrens et al, 2002; Eferl et al, 2003), also plays crucial roles in regulating the functions of macrophages (Hannemann et al, 2017). In both cases, c-Jun is necessary for activation of appropriate target genes which regulate the proper functioning of the cells. Using c-Jun deficiency as a means to functionally alter these cell types, we show that a coordinated response between hepatocytes and macrophages is necessary to regulate the liver fibrotic process (Fig 5F).

We demonstrate here that c-Jun acts as a pro-fibrogenic factor in both hepatocytes and KCs during liver fibrosis mediated by CCl_4_. Yet, absence of c-Jun in hepatocytes alone or in combination with KCs results in opposite outcomes. Isolated hepatocytes from both cohorts of mice exhibit a defect in cytokine gene expression, suggestive of a non-cell autonomous mechanism that contributes to enhanced fibrosis when c-Jun is deleted in hepatocytes alone. This is attributable to elevated macrophage numbers in the livers of the c-jun^f/f^;Alb-cre mice. Consistently, depletion of macrophages in these mice led to the reversal of the phenotype, confirming a compensatory effect of the macrophages during liver fibrosis when hepatocyte functions are specifically affected. These findings further demonstrate that an orchestrated coordination between hepatocytes and macrophages is crucial in regulating the fibrotic process. On the other hand, deletion of *c-jun* in KCs also led to decreased fibrosis, reiterating a pro-fibrogenic role for KCs. However, deletion of *c-jun* in KCs, which are a minor population in the liver compared with hepatocytes, did not appear to lead to any significant compensation by hepatocytes. In this regard, it is interesting to note that the magnitude of decrease in the expression of cytokine genes was significantly greater than the degree of fibrosis as ascertained by Sirius red staining when *c-jun* was deleted in KCs (Fig 5A, B, and E), indicating that the impact of defects in the minor KC population on overall liver fibrosis is relatively minimal. Not surprisingly, deletion of *c-jun* in both these cell types led to reduced fibrosis, consistent with their pro-fibrogenic role.

A noteworthy point is that we had used LysM-cre strain for deletion of *c-jun* in the KCs. LysM-cre mice have been extensively used for deletion in macrophages, and in particular, KCs in liver disease contexts (Greenhalgh et al, 2015; Beattie et al, 2016; Shi et al, 2018; Puchalska et al, 2019). However, they also delete in granulocytes (Shi et al, 2018), and the role of c-Jun in granulocytes have yet to be explored. Nevertheless, our data using chemical depletion of macrophages in the c-jun^f/f;^Alb-cre mice showed a reversal of the fibrotic phenotype, corroborating with the LysM-cre data and alludes strongly to a role for c-Jun in the KCs in contributing to liver fibrosis.

The phenomenon of compensatory increase in macrophages in the liver appears to be conserved with respect to liver damage. Absence of Egfr signaling in hepatocytes alone led to increased HCC formation induced by diethylnitrosamine/phenobarbital because of the compensatory increase in Egfr signaling in KCs (Lanaya et al, 2014). By contrast, HCC rates were reduced when *Egfr* was deleted in both hepatocytes and KCs (using the Mx-cre mice), or in KC cells alone (LysM-cre). Similarly, deletion of *IKK*β in hepatocytes alone led to increased hepatocarcinogenesis induced by dietheylnitrosamine (DEN), which was reversed upon *IKK*β deletion using Mx-cre mice (Maeda et al, 2005). Altogether, our data are consistent with previous studies of liver carcinogenesis and supports the notion that an orchestrated cross-talk between KCs and hepatocytes is required to regulate the liver fibrotic process.

A recent study that was published while our manuscript was under review also explored the role of c-Jun in non-alcoholic steotohepatitis (NASH) induced by methionine–choline–deficient diet (MCDD), which leads to liver fibrosis (Schulien et al, 2019). The conclusions of that study were also broadly similar to our work although with a different mechanistic basis. In essence, the MCDD-induced fibrosis and NASH also affects adipogenesis, unlike the CCl_4_-induced liver fibrosis. This is reflected in the different causal mechanisms that are predominant in these models. In the MCDD model, c-Jun has been suggested to regulate the expression of Opn and its receptor CD44 in non-parenchymal cells through the ductular reaction, leading to increased fibrosis in the liver-specific knockout. There was, however, no increase in F4/80+ cells in the Alb-cre model (rather, there was a decrease). By contrast, we see a significant increase in the numbers of F4/80+ cells in the Alb-cre knockout model, which has led us to deplete macrophages chemically and delete *c-jun* in macrophages using the LysM-Cre model. Furthermore, the MCDD model leads to increased cell death in the livers of the liver-specific knockout mice, which was reduced in the MX-cre model. However, in our CCl_4_ model, we do not see any difference in cell death in the Mx-cre model, again highlighting the mechanistic differences attributable to c-Jun deficiency in the different models. Altogether, although both studies have broadly come to similar conclusions, the various differences highlighted above complement both studies which together demonstrate the similarities in the broad principles of liver fibrosis regulation by c-Jun.

Our study has further explored the role of c-Jun in the fibrosis resolution process, which revealed that c-Jun expression was maintained significantly in the liver even 8 wk post-removal of the fibrotic stimulation. However, deletion of *c-jun* during this phase had no marked effects on the resolution rate or on the changes in any of the molecular markers, indicating that c-Jun expression is not critical for the recovery phase when the fibrotic stimuli is removed. This prompts us to speculate that the levels of c-Jun might be maintained as a provisionary measure for the rapid re-activation of the fibrotic process in the event that the fibrotic stimuli re-emerges, an hypothesis that requires future investigation.

Many molecular pathways, including several AP-1 pathway members have been shown to be active in and causal to fibrosis (Smart et al, 2006; Omenetti & Diehl, 2011; Wernig et al, 2017), and therapeutic interventions to target them are being explored. One caveat the current study highlights is the complexity of the systems and the interplay of cell types within an organ that is critical in controlling the collective eventual outcome, which would likely be similar for other signaling modules. Hence, it is important to understand the functions of the major signaling pathways in multiple tissues and cell types within them, before embarking on efforts to target them. Thus, any molecular therapeutic intervention requires judicious interrogation so as not to perturb the balance between cell types.

In summary, our study demonstrates yet another level of complexity of cellular signaling, using c-Jun as a model, in maintaining liver homeostasis during fibrosis. This work highlights the tight regulation that is maintained among cell types, which dictates the extent of fibrosis. It is likely that such a paradigm might be operative in other organs, and in other pathological conditions such as those that lead to wound healing. Hence, careful consideration ought to be given when using molecularly driven drugs.

## Materials and Methods

### Mouse breeding, induction of fibrosis, and perfusion

Mouse strains used in the study are as follows: c-jun^f/f^ (Behrens et al, 2002); Alb-creER; LysM-cre; and Mx-cre (Lanaya et al, 2014). All mice were bred, housed, and used for experiments with the approval and in accordance with the guidelines of the SingHealth’s Animal Care and Use Committee.

6-wk-old mice were treated twice a week with olive oil or carbon tetrachloride (CCl_4_) (1 μl per gram body weight [gbw] diluted in olive oil) by i.p. injection for 8 wk to induce fibrosis. Alb-creER (i.e., Alb) mice were in addition treated with tamoxifen (1 mg per mouse) by oral gavage to activate the Cre-recombinase (as per schedule shown in Fig 1B). Mice expressing Mx-cre were administered with Poly I:C (13 μg/gbw) by i.p. injection to induce Cre expression 1 wk before CCl_4_ injection. 10 μg/gbw of gadolinium chloride (GdCl_3_) or PBS was injected i.v. once a week for 8 wk during the course of CCl_4_ injection, in the indicated studies.

For regression studies, 6-wk-old mice were treated twice a week with olive oil or CCl_4_ as described for 6 wk to induce fibrosis. Mice expressing Mx-cre were administered with Poly I:C (13 μg/gbw) by i.p. injection to induce Cre expression 5 wk after the start of the CCl_4_ injection. Mice were kept for an additional of 4 or 8 wk after the last CCl_4_ injection and analyzed thereafter.

All mice were euthanized 72 h after the last CCl_4_ injection and their livers were excised, frozen, or formalin-fixed for further analysis.

**Table:** Genotyping was performed using the following primers, with the expected size of PCR products are indicated in the table below:

Primers	Sequence	Expected PCR size (bp)
c-Jun lox5 (For)	CTCATACCAGTTCGCACAGGC	
c-Jun lox6 (Rev 1)	CGCTAGCACTCACGTTGGTAG	350
c-Jun flox2 (Rev 2)	CAGGGCGTTGTGTCACTGAGCT	4,000/600
Cre internal Ctrl (For)	CTAGGCCACAGAATTGAAAGATCT	324
Cre internal Ctrl (Rev)	GTAGGTGGAAATTCTAGCATCATCC	
Cre transgene (For)	TCCAATTTACTGACCGTACACCAA	500
Cre transgene (Rev)	CCTGATCCTGGCAATTTCGGCTA	

For quantification on the efficiency of *c-jun* deletion, image J was used to measure the intensity of bands. These values were then converted as a percentage of the floxxed band over the total floxxed and unfloxxed bands.

For perfusion and collection of cells, mice were anaesthetized by i.p. injection of 5 μg/gbw Diazepam and 50 μg/gbw Ketamine. Mice were then perfused from inferior vena cava and out from the hepatic portal vein. 3.7 U of collagenase D was used to flush through the liver before ex-vivo digestion using the same solution for 5 min. Cells were then passed through a 70 μM strainer, and centrifuged at 50*g* for 10 min to isolate out purified hepatocytes (Severgnini et al, 2012).

### Quantitative gene expression assay

Total RNA was prepared and used to synthesize cDNA using SuperScript II reverse transcriptase, as described (Subramanian et al, 2015). qRT-PCR was performed using gene-specific primers (Table S1) and QuantiFast SYBR Green PCR Kit in Rotor-Gene Q real-time PCR machine (QIAGEN) and ViiA7 real-time PCR system (Thermo Fisher Scientific) according to manufacturer’s instructions, and relative gene expression was normalized with *Gapdh* expression. 2−∆∆Ct was used to compare the transcriptional differences. Cycle threshold (Ct) values of various genes were plotted against Ct values of *Gapdh* to determine a ratio between different treatment groups.

Table S1 Primers used for qRT-PCR

### Immunoblot analysis

Small pieces of liver tissues were lysed and ∼80 μg of proteins were used and analyzed by immunoblot. Antibodies against c-Jun (9165; CST), Acta-2 (M0851; Dako), Desmin (14026; Santa Cruz), Vimentin (5741; CST), TGF-β1 (130348; Santa Cruz), TNF-α (52746; Santa Cruz), Gapdh (25778; Santa Cruz), p-JNK (9255; CST), and t-JNK (7345; Santa Cruz) were used in this work. Multiple gels were run with the same set of lysates from each cohort of mice to be probed with the various antibodies, with one or two blots being used for assessment of loading using the anti-Gapdh antibody.

### Histological and related analysis

Liver tissues (all lobes) were fixed in 10% formalin overnight, and then dehydrated and embedded in paraffin blocks, which were sectioned at a thickness of 5 μm. For Sirius red staining, sections were stained with hematoxylin (Weigert’s) for 8 min, washed with running tap water for 10 min, followed by incubation with 0.1% (wt/vol) Sirius red diluted in picric acid solution for 1 h. The slides were then rinsed in two quick changes of 0.5% (vol/vol) acetic acid to remove unbound dye. For immunostaining, sections were first heated in sodium citrate buffer to retrieve antigen. Sections were then blocked in 10% FBS blocking buffer followed by incubation with the following primary antibodies: anti-c-Jun (60A8; Cell Signaling), anti-F4/80 (SP115; Thermo Fisher Scientific), and anti-Ki67 (15580; Abcam), before quenching endogenous peroxidase using 3% hydrogen peroxide. TUNEL staining was performed as per manufacturer’s recommendations using the apoptosis detection kit (206386; Abcam). To quantify staining, 20 randomly taken images of 10× fields per section were evaluated by Meta Imaging Series (Molecular Devices) software.

A small portion of the liver was homogenized in ice cold NaCl buffer. The supernatant was collected, normalized before subjected to AST/ALT measurements using an AST/ALT Assay kit (Nanjing Jiancheng Bioengineering Institute) according to the supplier’s protocol.

### Statistical analysis

All data are presented as mean ± SD. The results were analyzed by unpaired *t* test, ANOVA or by two-way ANOVA, as appropriate. Statistical calculation was performed using GraphPad Prism software (5.0). The animal numbers used for each experiment are indicated in each of the figure legends. *P*-values less than 0.05 were considered to be statistically significant.

## Supplementary Material

Reviewer comments
