# Supplementary material for: Functional interaction between macrophages and hepatocytes dictate the outcome of liver fibrosis
Source: Life Sci Alliance. 2021 Jan 29;4(4):e202000803. doi: 10.26508/lsa.202000803 (PMC7893818; doi:10.26508/lsa.202000803)

Figure 1C left

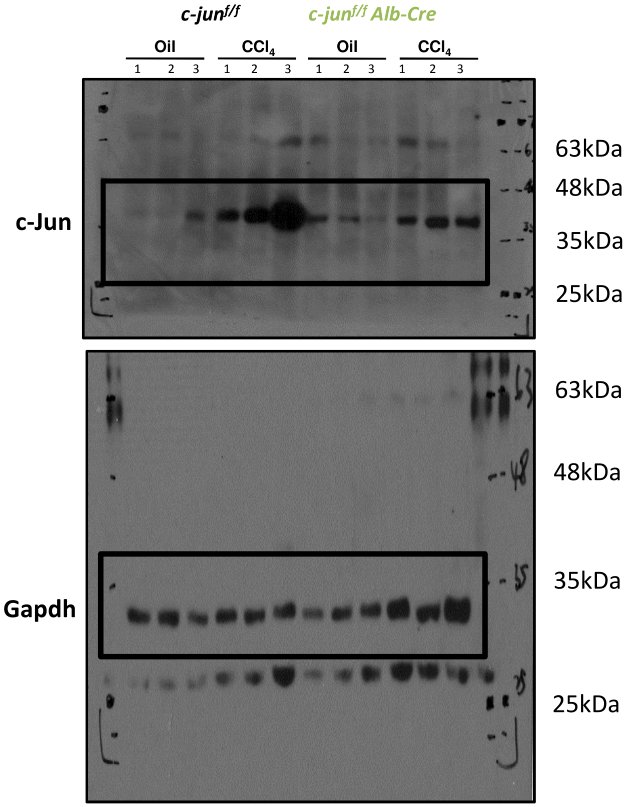

Figure 1C right

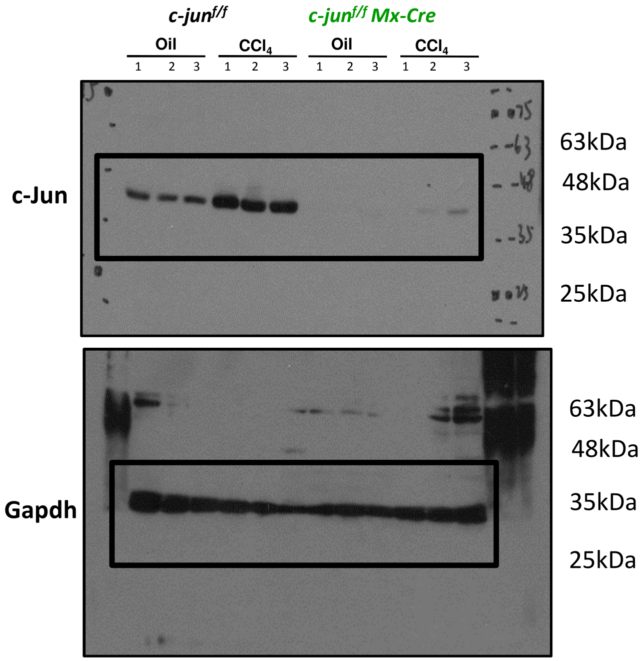

Figure 2F left

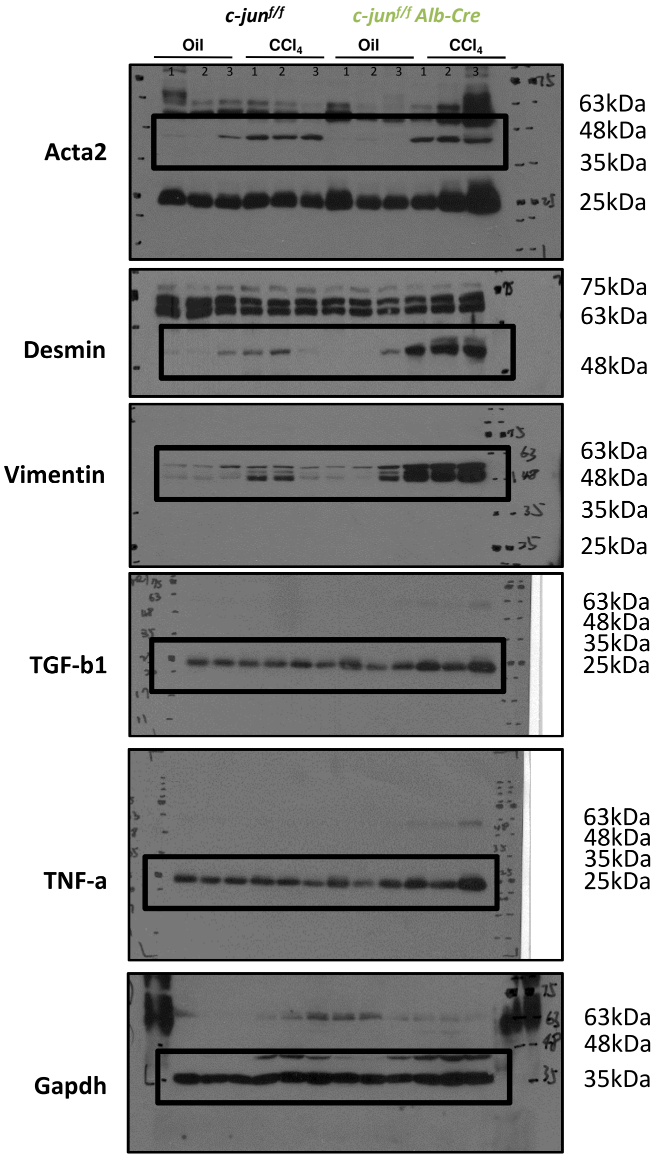

Figure 2F right

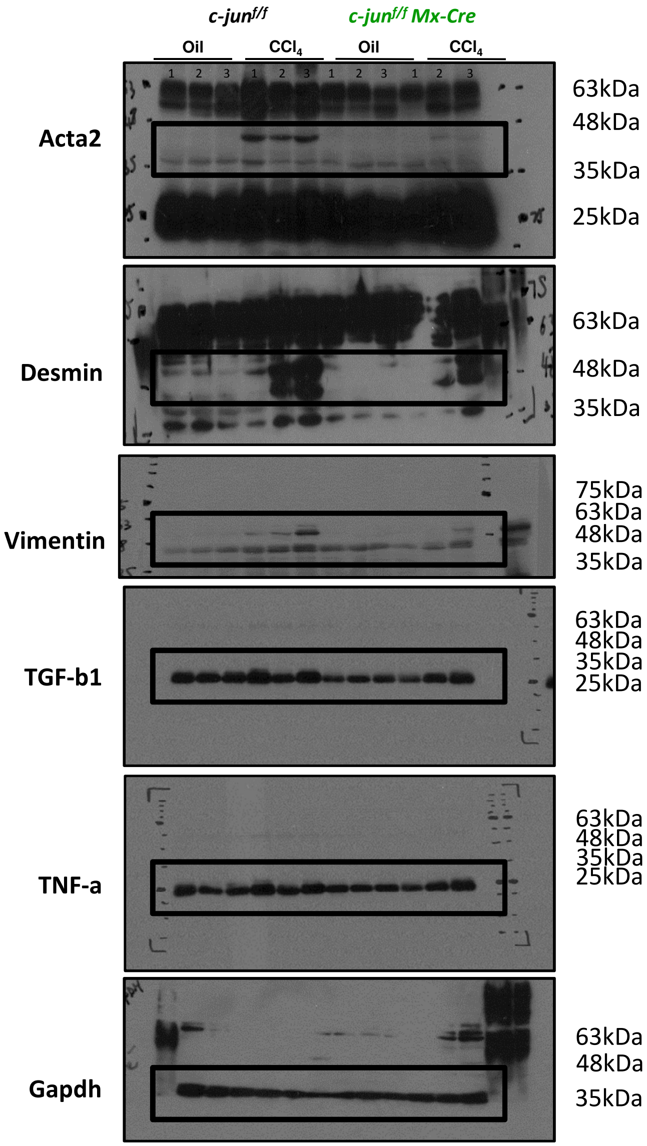

Figure 5E

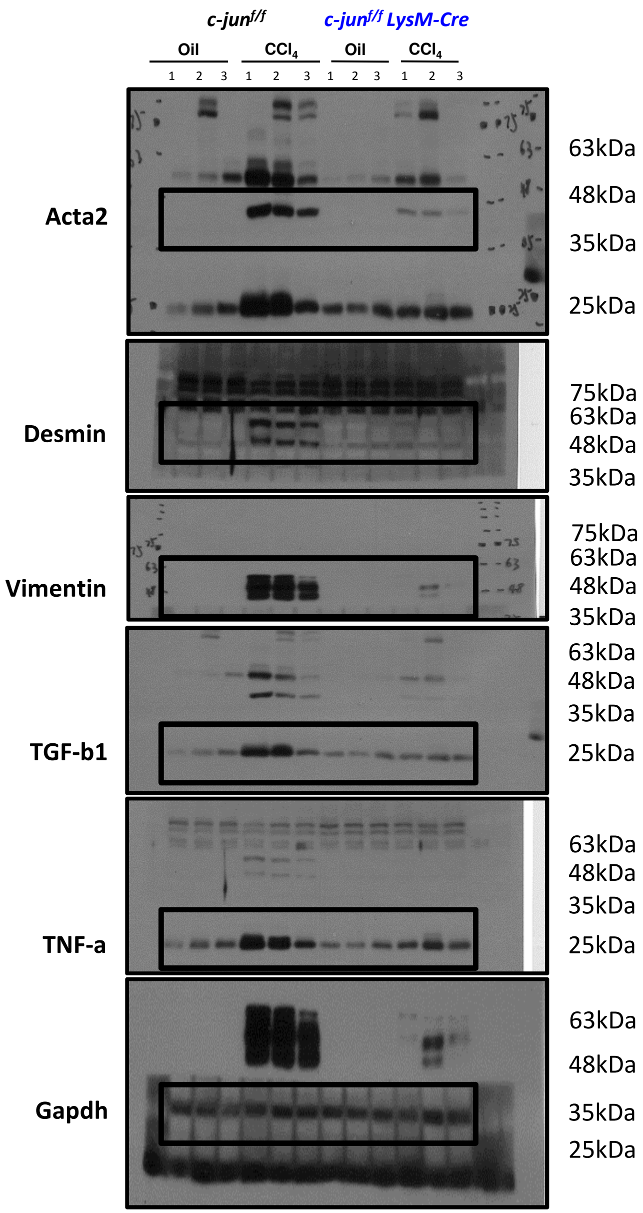

c-jun f/f
c-jun f/f LysM-Cre
Oil
CCl4
Oil
CCl4
1 2 3 1 2 3 1 2 3 1 2 3
Acta2
63kDa
48kDa
35kDa
25kDa
Desmin
75kDa
63kDa
48kDa
35kDa
Vimentin
75kDa
63kDa
48kDa
35kDa
TGF-b1
63kDa
48kDa
35kDa
25kDa
TNF-a
63kDa
48kDa
35kDa
25kDa
Gapdh
63kDa
48kDa
35kDa
25kDa

Figure 6B

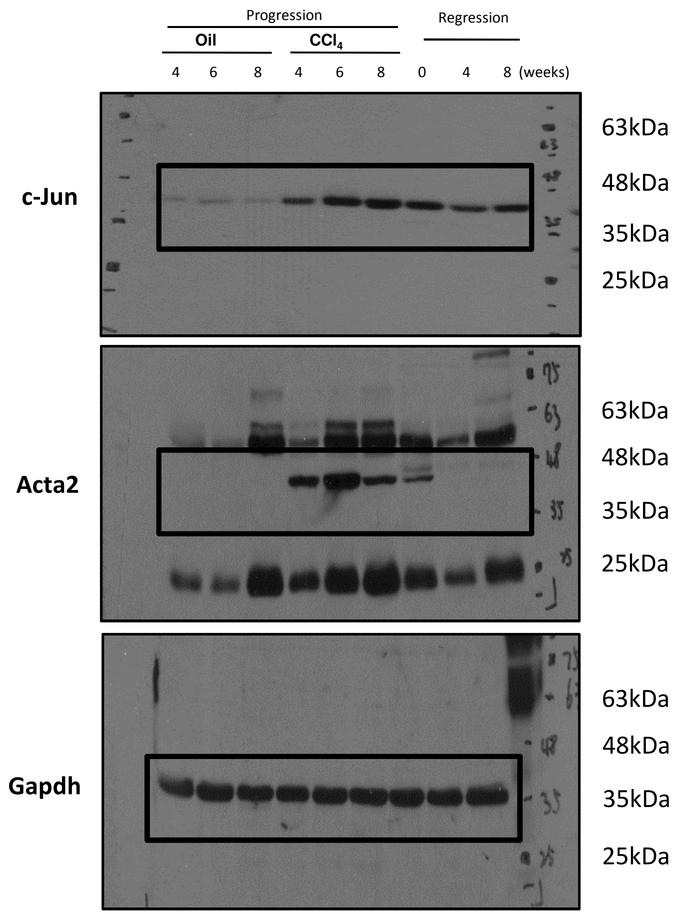
Progression
Regression
Oil
CCl_4
4 6 8 4 6 8 0 4 8 (weeks)
c-Jun
Acta2
Gapdh
63kDa
48kDa
35kDa
25kDa
63kDa
48kDa
35kDa
25kDa
63kDa
48kDa
35kDa
25kDa

Suppl Figure 1D left

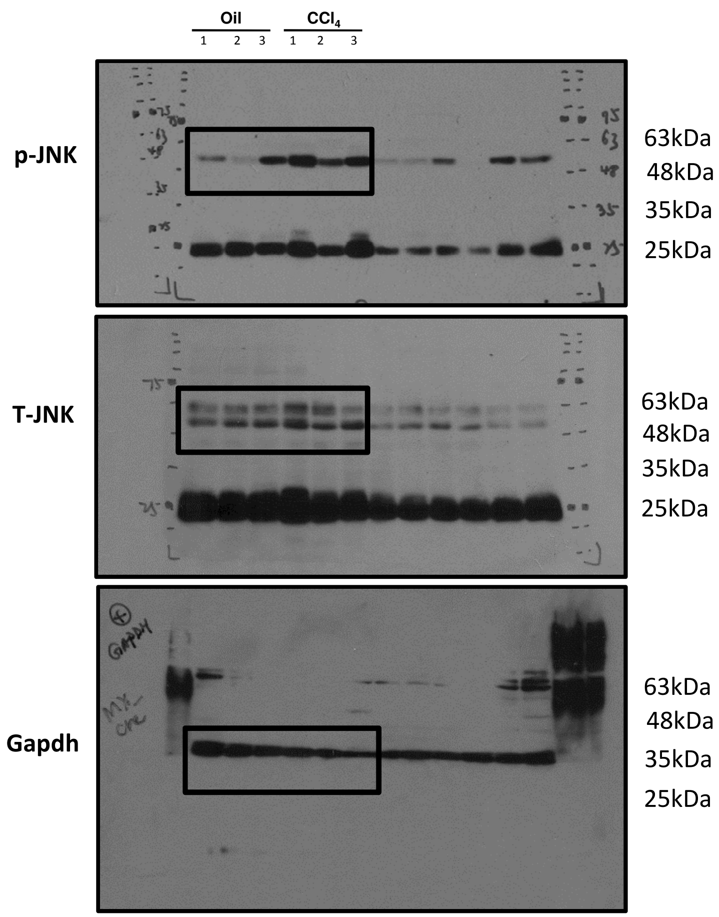

Suppl Figure 1D right

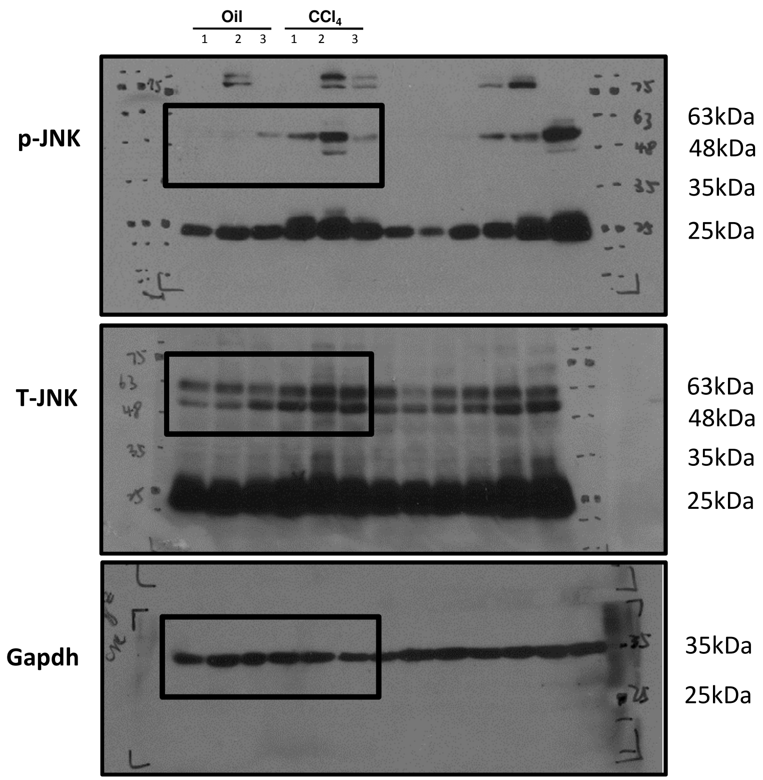

Supplement: Supplementary file 1 [file LSA-2020-00803_SdataF1_F2_F5_F6_FS1.pdf]
