# Supplementary material for: Functional interaction between macrophages and hepatocytes dictate the outcome of liver fibrosis
Source: Life Sci Alliance. 2021 Jan 29;4(4):e202000803. doi: 10.26508/lsa.202000803 (PMC7893818; doi:10.26508/lsa.202000803)
Supplement: Supplementary file 2 [file LSA-2020-00803_TableS1.docx]

**Supplementary Table 1. Primers used for qRT-PCR.**

| Primers | Sequence |
| --- | --- |
| mGapdh_For | ATCTTCTTGTGCAGTGCCAG |
| mGapdh_Rev | GTAGTTGAGGTCAATGAAGG |
| mc-Jun_For | CCTTCTACGACGATGCCCTC |
| mc-Jun_Rev | GGTTCAAGGTCATGCTCTGTTT |
| mActa2_For | GAGGCACCACTGAACCCTAA |
| mActa2_Rev | GTTGTACGTCCAGAGGCATAGA |
| mDesmin_For | TACACCTGCGAGATTGATGC |
| mDesmin_Rev | ACATCCAAGGCCATCTTCAC |
| mVimentin_For | TCTCTGGCACGTCTTGACC |
| mVimentin_Rev | GCCACGCTTTCATACTGCT |
| mTgfb1_For | CCATTGCTGTCCCGTGCAGA |
| mTgfb1_Rev | TTGGTTCAGCCACTGCCGTA |
| mTnf-α_For | CCTGTAGCCCACGTCGTA |
| mTnf-α_Rev | GGGAGTAGACAAGGTACA |
| mIL1b_For | TGCACTACAGGCTCCGAGA |
| mIL1b_Rev | AGGCCACAGGTATTTTGTCGTT |
| mPdgfb_For | CATCCGCTCCTTTGATGA |
| mPdgfb_Rev | GTGCTCGGGTCATGTTCA |
| mCol1a1_For | GCTCCTCTTAGGGGCCACT |
| mCol1a1_Rev | CCACGTCTCACCATTGGGG |
| mCol1a2_For | AGGCCCAACCTGTAAACACC |
| mCol1a2_Rev | GAGGACACCCCTTCTACGTT |
| mCol3a1_For | CTGTAACATGGAAACTGGGGAAA |
| mCol3a1_Rev | CCATAGCTGAACTGAAAACCACC |
| mTimp1_For | TGCAGCTTCTTGGTTCCCT |
| mTimp1_Rev | TAGTCCTCAGAGCCCACGA |
| mTimp2_For | TCAGAGCCAAAGCAGTGAGC |
| mTimp2_Rev | GCCGTGTAGATAAACTCGATGTC |
| mMMP8_For | TCTTCCTCCACACACAGCTTG |
| mMMP8_Rev | CTGCAACCATCGTGGCATTC |
| mMMP13_For | CTTCTTCTTGTTGAGCTGGACTC |
| mMMP13_Rev | CTGTGGAGGTCACTGTAGACT |
