# Supplementary material for: Functional interaction between macrophages and hepatocytes dictate the outcome of liver fibrosis
Source: Life Sci Alliance. 2021 Jan 29;4(4):e202000803. doi: 10.26508/lsa.202000803 (PMC7893818; doi:10.26508/lsa.202000803)
Supplement: Reviewer comments [file LSA-2020-00803_review_history.pdf]

Life Science Alliance

Functional interaction between macrophages and hepatocytes dictate the outcome of liver fibrosis

Min Xie, Ren Hui Chia, Dan Li, Xueting Teo, Christian Krueger and Kanaga Sabapathy
DOI: https://doi.org/10.26508/lsa.202000803

Corresponding author(s): Kanaga Sabapathy, National Cancer Centre

Review Timeline:

Submission Date:	2020-06-01
Editorial Decision:	2020-07-14
Revision Received:	2020-11-16
Editorial Decision:	2021-01-05
Revision Received:	2021-01-14
Accepted:	2021-01-15

Scientific Editor: Shachi Bhatt

Transaction Report:

(Note: With the exception of the correction of typographical or spelling errors that could be a source of ambiguity, letters and reports are not edited. The original formatting of letters and referee reports may not be reflected in this compilation.)

No full Peer Review Process File is available with this article, as the authors have chosen not to make the review process public in this case.

July 14, 2020

Re: Life Science Alliance manuscript #LSA-2020-00803-T

Prof. Kanaga Sabapathy
National Cancer Centre
Cellular adn Molecular Research
11, Hospital Drive
Singapore 169610
Singapore

Dear Dr. Sabapathy,

Thank you for submitting your manuscript entitled "Functional interaction between macrophages and hepatocytes dictate outcome of liver fibrosis" to Life Science Alliance. The manuscript was assessed by expert reviewers, whose comments are appended to this letter.

The referees appreciate the analysis, but they also raise significant concerns that need to be addressed for publication in this journal. Most importantly, in its current state, as far as we could tell and according to referee #3, the dataset adds limited information over Schulien et al 2019 (this issue was not discussed in detail in the paper or cover letter where any additional advances should have been emphasized). Therefore, it is necessary to develop the current dataset to add sufficient value to the scientific community to warrant publication in Life Science Alliance. We would be open to discuss a suitable revision in the future.

- Referees #1 and 3 would like to gain insight into the role of c-Jun in fibrosis resolution.
- Referee #2 would like to get more insight as to which pathway upstream of c-Jun is activated.
- Referee #3 requests transcriptomic analysis on the isolated cells.
- Referees request additional controls, which need to be provided (referee #1, referee #2 points 1 and 4, referee #3).

If you cannot address point 5 of referee #2, this will not preclude from publication.

Should you be able to address these points and willing to expand the dataset over Schulien et al 2019 as mentioned above, we would be happy to consider a revised manuscript. Please note that we will need strong support from the referees on the revised version.
In our view these revisions should typically be achievable in around 3 months. However, we are aware that many laboratories cannot function fully during the current COVID-19/SARS-CoV-2 pandemic and therefore encourage you to take the time necessary to revise the manuscript to the extend requested above. We will extend our 'scoping protection policy' to the full revision period required. If you do see another paper with related content published elsewhere, nonetheless contact me immediately so that we can discuss the best way to proceed.

To upload the revised version of your manuscript, please log in to your account: https://lsa.msubmit.net/cgi-bin/main.plex
You will be guided to complete the submission of your revised manuscript and to fill in all necessary information. Please get in touch in case you do not know or remember your login name.

We would be happy to discuss the individual revision points further with you should this be helpful.

While you are revising your manuscript, please also attend to the below editorial points to help expedite the publication of your manuscript. Please direct any editorial questions to the journal office.

Please note that papers are generally considered through only one revision cycle, so strong support from the referees on the revised version is needed for acceptance.

When submitting the revision, please include a letter addressing the reviewers' comments point by point.

We hope that the comments below will prove constructive as your work progresses.

Thank you for this interesting contribution to Life Science Alliance. We are looking forward to receiving your revised manuscript.

Sincerely,

Reilly Lorenz
Editorial Office Life Science Alliance
Meyerhofstr. 1
69117 Heidelberg, Germany
t +49 6221 8891 414
e contact@life-science-alliance.org
www.life-science-alliance.org

A. THESE ITEMS ARE REQUIRED FOR REVISIONS

-- A letter addressing the reviewers' comments point by point.

-- An editable version of the final text (.DOC or .DOCX) is needed for copyediting (no PDFs).

-- High-resolution figure, supplementary figure and video files uploaded as individual files: See our detailed guidelines for preparing your production-ready images, http://www.life-science-alliance.org/authors

-- Summary blurb (enter in submission system): A short text summarizing in a single sentence the study (max. 200 characters including spaces). This text is used in conjunction with the titles of papers, hence should be informative and complementary to the title and running title. It should describe the context and significance of the findings for a general readership; it should be written in the present tense and refer to the work in the third person. Author names should not be mentioned.

B. MANUSCRIPT ORGANIZATION AND FORMATTING:

Full guidelines are available on our Instructions for Authors page, http://www.life-science-alliance.org/authors

We encourage our authors to provide original source data, particularly uncropped/-processed electrophoretic blots and spreadsheets for the main figures of the manuscript. If you would like to add source data, we would welcome one PDF/Excel-file per figure for this information. These files will be linked online as supplementary "Source Data" files.

IMPORTANT: It is Life Science Alliance policy that if requested, original data images must be made available. Failure to provide original images upon request will result in unavoidable delays in publication. Please ensure that you have access to all original microscopy and blot data images before submitting your revision.

January 5, 2021

RE: Life Science Alliance Manuscript #LSA-2020-00803-TR

Prof. Kanaga Sabapathy
National Cancer Centre
Cellular and Molecular Research
11, Hospital Crescent
Singapore 169610
Singapore

Dear Dr. Sabapathy,

Thank you for submitting your revised manuscript entitled "Functional interaction between macrophages and hepatocytes dictate the outcome of liver fibrosis". We would be happy to publish your paper in Life Science Alliance pending final revisions necessary to meet our formatting guidelines.

We apologize for this extensive delay in getting back to you. As you will note from the reviewers' comments section below, we were unable to secure the comments of Reviewer 2 and 3 despite our several tries, also the cause for this extended delay. However, we are pleased to say that we have gone through the revised manuscript and the rebuttal in detail and deemed that you have sufficiently addressed the concerns of all the reviewers. The manuscript is thus publishable in Life Science Alliance with minimal formatting changes.

Along with the points listed below, we would also appreciate if you can attend to the following:
-please upload both your main and supplementary figures as single files
-please change your EV figures to Supplementary figures - both in the figure legends and in the figure callouts in the main manuscript text
-please re-write the Fig 2 legend to explain the individual panels better
-Fig 2 legend: do they have to explain individual panels better?
-please add the legends for panel C-E in the legend for Figure EV4
-please provide the original uncropped images for the Gapdh blots shown in both Figure 2F and Figure 1C. Are these the same blot? It is our policy to not use the same blot; if this is not possible to get a new blot image, we request you specify this in the figure legends
-please add scale bars for Fig 6 D,E and Figure EV4-A

If you are planning a press release on your work, please inform us immediately to allow informing our production team and scheduling a release date.

To upload the final version of your manuscript, please log in to your account: https://lsa.msubmit.net/cgi-bin/main.plex
You will be guided to complete the submission of your revised manuscript and to fill in all necessary information. Please get in touch in case you do not know or remember your login name.

To avoid unnecessary delays in the acceptance and publication of your paper, please read the following information carefully.

A. FINAL FILES:

These items are required for acceptance.

-- An editable version of the final text (.DOC or .DOCX) is needed for copyediting (no PDFs).

-- High-resolution figure, supplementary figure and video files uploaded as individual files: See our detailed guidelines for preparing your production-ready images, https://www.life-science-alliance.org/authors

-- Summary blurb (enter in submission system): A short text summarizing in a single sentence the study (max. 200 characters including spaces). This text is used in conjunction with the titles of papers, hence should be informative and complementary to the title. It should describe the context and significance of the findings for a general readership; it should be written in the present tense and refer to the work in the third person. Author names should not be mentioned.

B. MANUSCRIPT ORGANIZATION AND FORMATTING:

Full guidelines are available on our Instructions for Authors page, https://www.life-science-alliance.org/authors

We encourage our authors to provide original source data, particularly uncropped/-processed electrophoretic blots and spreadsheets for the main figures of the manuscript. If you would like to add source data, we would welcome one PDF/Excel-file per figure for this information. These files will be linked online as supplementary "Source Data" files.

Submission of a paper that does not conform to Life Science Alliance guidelines will delay the acceptance of your manuscript.

It is Life Science Alliance policy that if requested, original data images must be made available to the editors. Failure to provide original images upon request will result in unavoidable delays in publication. Please ensure that you have access to all original data images prior to final submission.

The license to publish form must be signed before your manuscript can be sent to production. A link to the electronic license to publish form will be sent to the corresponding author only. Please take a moment to check your funder requirements.

Reviews, decision letters, and point-by-point responses associated with peer-review at Life Science Alliance will be published online, alongside the manuscript. If you do want to opt out of having the reviewer reports and your point-by-point responses displayed, please let us know immediately.

Thank you for your attention to these final processing requirements. Please revise and format the manuscript and upload materials within 7 days.

Thank you for this interesting contribution, we look forward to publishing your paper in Life Science Alliance.

Sincerely,

Shachi Bhatt, Ph.D.
Executive Editor
Life Science Alliance
https://www.lsajournal.org/
Tweet @SciBhatt @LSAjournal

January 15, 2021

RE: Life Science Alliance Manuscript #LSA-2020-00803-TRR

Prof. Kanaga Sabapathy
National Cancer Centre
Cellular and Molecular Research
11, Hospital Crescent
Singapore 169610
Singapore

Dear Dr. Sabapathy,

Thank you for submitting your Research Article entitled "Functional interaction between macrophages and hepatocytes dictate the outcome of liver fibrosis". It is a pleasure to let you know that your manuscript is now accepted for publication in Life Science Alliance. Congratulations on this interesting work.

The final published version of your manuscript will be deposited by us to PubMed Central upon online publication.

Reviews, decision letters, and point-by-point responses associated with peer-review at Life Science Alliance will be published online, alongside the manuscript. If you do want to opt out of having the reviewer reports and your point-by-point responses displayed, please let us know immediately.

IMPORTANT: If you will be unreachable at any time, please provide us with the email address of an alternate author. Failure to respond to routine queries may lead to unavoidable delays in publication.

Scheduling details will be available from our production department. You will receive proofs shortly before the publication date. Only essential corrections can be made at the proof stage so if there are any minor final changes you wish to make to the manuscript, please let the journal office know now.

DISTRIBUTION OF MATERIALS:
Authors are required to distribute freely any materials used in experiments published in Life Science Alliance. Authors are encouraged to deposit materials used in their studies to the appropriate repositories for distribution to researchers.

You can contact the journal office with any questions, contact@life-science-alliance.org

Again, congratulations on a very nice paper. I hope you found the review process to be constructive and are pleased with how the manuscript was handled editorially. We look forward to future exciting submissions from your lab.

Sincerely,

Shachi Bhatt, Ph.D.
Executive Editor
Life Science Alliance
https://www.lsajournal.org/
Tweet @SciBhatt @LSAjournal
